# Serum metabolic traits reveal therapeutic toxicities and responses of neoadjuvant chemoradiotherapy in patients with rectal cancer

**DOI:** 10.1038/s41467-022-35511-y

**Published:** 2022-12-17

**Authors:** Hongmiao Wang, Huixun Jia, Yang Gao, Haosong Zhang, Jin Fan, Lijie Zhang, Fandong Ren, Yandong Yin, Yuping Cai, Ji Zhu, Zheng-Jiang Zhu

**Affiliations:** 1 Interdisciplinary Research Center on Biology and Chemistry, Shanghai Institute of Organic Chemistry, Chinese Academy of Sciences, Shanghai, 200032 China; 2 University of Chinese Academy of Sciences, Beijing, 100049 China; 3 Department of Ophthalmology, Shanghai General Hospital, Shanghai Jiao Tong University School of Medicine, Shanghai, 200080 China; 4 Department of Biostatistics, Fudan University Shanghai Cancer Center, Shanghai, 200032 China; 5 Cancer Hospital of the University of Chinese Academy of Sciences (Zhejiang Cancer Hospital), Hangzhou, 310005 China; 6 Institute of Cancer and Basic Medicine, Chinese Academy of Sciences, Hangzhou, 310000 China; 7 Zhejiang Key Laboratory of Radiation Oncology, Hangzhou, 310000 China; 8 Department of Radiation Oncology, Fudan University Shanghai Cancer Center, Shanghai, 200032 China; 9 Department of Oncology, Shanghai Medical College, Fudan University, Shanghai, 200031 China; 10 Shanghai Key Laboratory of Radiation Oncology, Shanghai, 200032 China; 11 Shanghai Key Laboratory of Aging Studies, Shanghai, 201210 China

**Keywords:** Metabolomics, Colorectal cancer, Mass spectrometry

## Abstract

Neoadjuvant chemoradiotherapy (nCRT) has become the standard treatment for patients with locally advanced rectal cancer (LARC). Therapeutic efficacy of nCRT is significantly affected by treatment-induced diarrhea and hematologic toxicities. Metabolic alternations in cancer therapy are key determinants to therapeutic toxicities and responses, but exploration in large-scale clinical studies remains limited. Here, we analyze 743 serum samples from 165 LARC patients recruited in a phase III clinical study using untargeted metabolomics and identify responsive metabolic traits over the course of nCRT. Pre-therapeutic serum metabolites successfully predict the chances of diarrhea and hematologic toxicities during nCRT. Particularly, levels of acyl carnitines are linked to sex disparity in nCRT-induced diarrhea. Finally, we show that differences in phenylalanine metabolism and essential amino acid metabolism may underlie distinct therapeutic responses of nCRT. This study illustrates the metabolic dynamics over the course of nCRT and provides potential to guide personalized nCRT treatment using responsive metabolic traits.

## Abstract (web summary)

Neoadjuvant chemoradiotherapy is the standard treatment for patients with locally advanced rectal cancer, however, response can be limited by development of toxicities. Here, the authors conducted metabolomics on patients with locally advanced rectal cancer enrolled in a phase III clinical study and identify serum metabolites associated with treatment response.

## Introduction

Colorectal cancer (CRC) is the third most commonly diagnosed cancer and the fourth leading cause of cancer-related deaths worldwide^1^. Up to 15% CRC patients had locally advanced rectal cancer (LARC) at the time of diagnosis, which is of great concern as patients with LARC are at high risk for postoperative local recurrence and distant metastasis^2–4^. Neoadjuvant chemoradiotherapy (nCRT) followed by total mesorectal excision (TME) is the standard treatment for patients with LARC^5^. Patients with complete clinical response (cCR) after nCRT can follow the watch-and-wait strategy^6^. This strategy provides LARC patients with the opportunities to preserve the anus without radical surgery and improves life qualities. However, patients vary considerably in response to nCRT and individualized therapeutic strategies are therefore imperative needs for their treatments. Clinical statistics showed that only 10–35% patients can achieve pathological complete response (pCR)^7^. As a comparison, 40% to 45% patients present tumor regression with different grades, while the rest 20–30% have no responses to nCRT at all and such patients have to undergo total mesorectal excision surgery immediately^8,9^. In clinical practice, presurgical examinations including magnetic resonance imaging, endoscopy, and digital rectal examination have been used to evaluate the efficacy of nCRT treatment^10^. But these methods are insufficient to provide accurate evaluations, thereby impeding personalized therapy amid the nCRT treatment of LARC patients.

Therapeutic responses of LARC patients to nCRT treatment depend on many genetic and clinical factors. Over the course of nCRT treatment, LARC patients experience adverse effects such as leukopenia, neutropenia, and diarrhea^11–14^. The treatment-induced toxicities are responsible for cessation of treatment for patients who are unable to tolerate high dose of radio- and chemo- therapies. Therefore, adverse events are key determinants of nCRT therapeutic responses and ultimate clinical outcomes. For example, a multicenter and randomized phase III study showed that up to 20–36% LARC patients who received nCRT treatments presented with preoperative grade 3–4 toxic effects^14^. The treatment-related adverse events led to radiotherapy interruption in 7–10% patients, radiotherapy dose reduction in 3% patients, and concurrent chemotherapy dose reduction in 15–21% patients depending on the drugs used^14^. It is also evident that interpatient variability of adverse events is commonly present in LARC patients treated with nCRT. For example, grade 3–4 diarrhea, stomatitis, and alopecia were significantly more frequent in females than in males treated with FOLFIRI (a combination therapy consisting of leucovorin, fluorouracil, and irinotecan)^15^. But the reason for this sex-specific difference is unknown. In addition, LARC patients vary in the risk of severe neutropenia from the treatment of irinotecan based nCRT, which is suggested to be related in part to UGT1A1*28 (uridine diphosphate glucuronosyltransferase 1A1*28), a genetic variant that reduces the elimination of metabolic product of irinotecan^16,17^. Therefore, a variety of factors play critical roles in clinical outcomes for LARC patients with nCRT treatment. It is thus imperative to characterize the systemic effects of nCRT treatment and in particular to predict the toxic effects prior to therapy, with the ultimate goal of minimizing adverse effects and improving patient benefits.

Metabolomics provides comprehensive measurements of metabolites in biological systems and offers molecular insights towards pathological phenotypes^18,19^. It is widely recognized that metabolic dysregulation contributes to colorectal cancer, including through aberrant glycolysis, glutaminolysis, one-carbon metabolism, and fatty acid synthesis^20–22^. However, exploration of responsive metabolic traits in the context of neoadjuvant chemoradiotherapy remains limited. Though metabolism alterations induced by chemotherapy or radiotherapy have been reported in animal models^23,24^, metabolomics studies on large-scale clinical cohorts of LARC patients with nCRT treatment are rather limited^25,26^. In this work, we performed untargeted metabolomics for the CliClare study to reveal metabolic traits in response to therapeutic toxicities and efficacy of nCRT in patients with rectal cancer. The CliClare study is a multicenter, randomized, phase III clinical trial to evaluate the use of the *UGT1A1* genotype to guide the irinotecan dose when used in combination with capecitabine-based nCRT in LARC patients (ClinicalTrials.gov identifier: NCT02605265)^27^. We demonstrated that the pCR rate was significantly increased from 15% to 30% using the capecitabine and irinotecan-based treatment regime compared to capecitabine-based regime, however, increases in the frequency of grade 3–4 toxicities were also observed. Here, we analyzed serum samples (*n* = 743) from 165 LARC patients in CliClare study over their nCRT treatments, and identified responsive metabolic traits that were correlated with nCRT treatment. We further investigated the links between serum metabolic traits and adverse toxicity effects induced by nCRT, including diarrhea and hematologic toxicity. Particularly, we found that levels of acyl carnitines are linked to sex disparity in diarrhea induced by nCRT. Finally, we examined the metabolic alterations over the course of nCRT treatment and clinical outcomes, and discovered significant differences in gut microbiota related phenylalanine metabolism and essential amino acid metabolism between pCR and non-pCR patients. Altogether, our results reveal the metabolic dynamics over the course of nCRT treatment and provide great potentials to guide personalized nCRT treatment using responsive serum metabolic traits.

## Results

### Serum metabolic traits in response to nCRT in LARC patients

**Fig. 1:**
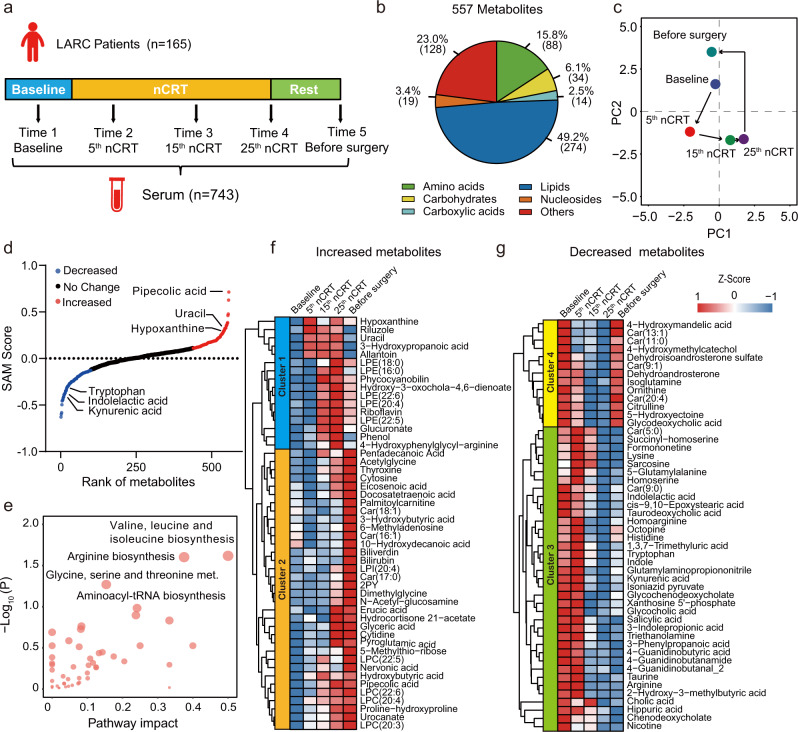
Serum metabolic traits in response to nCRT in LARC patients. **a** Overview of the clinical design of the metabolomics study. **b** Number of identified metabolites in serum samples and distributions of chemical classes. **c** Principal component analysis (PCA) shows significantly altered metabolic profiles over the course of nCRT. Each dot represents the averaged value from all patients. **d** Metabolites were significantly associated with the nCRT treatment analyzed by SAM (Significance Analysis of Microarrays; FDR adjusted *P* < 0.05). Red dots (*n* = 119) and blue dots (*n* = 100) represent metabolites that were increased and decreased, respectively, over the course of nCRT. The black dots represent unchanged metabolites. **e** Pathway **e**nrichment analysis using significantly changed metabolites (*n* = 219) associated with nCRT. (Hypergeometric test; *P* < 0.05). The pathway enrichment analysis was performed using MetaboAnalyst. **f**, **g** Hierarchical clustering analyses (HCA) using the top 50 increased (**f**) and top 50 decreased (**g**) metabolites over the course of nCRT.

In this study, we recruited 165 patients with clinical T3-4 and/or N + rectal cancer diagnosed from Fudan University Shanghai Cancer Center (FUSCC), Shanghai, China. All enrolled patients were treated with the standard neoadjuvant chemoradiotherapy (nCRT) protocol followed by total mesorectal excision surgery (see Methods section). The detailed information on the clinical cohort is listed in Supplementary Table 1. To investigate the metabolic dynamics over the course of nCRT, serum samples for each patient were collected at the following time points: before nCRT (Time 1), at the 5th fractions of nCRT (Time 2), at the 15th fractions of nCRT (Time 3), at the 25th fractions of nCRT (Time 4), and after the rest for two months and within 2 days before surgery (Time 5) (see Fig. 1a and Supplementary Fig. 1).

Comprehensive untargeted metabolomics analyses using hydrophilic interaction liquid chromatography-mass spectrometry (HILIC−MS) and reversed-phase liquid chromatography-mass spectrometry (RPLC−MS) were performed on serum samples from locally advanced rectal cancer (LARC) patients (*n* = 743 in total). In total, 557 metabolites were identified on the basis of the Metabolomics Standards Initiative (MSI)^28^ (Fig. 1b and Supplementary Data 1). As a result of the unsupervised principal component analysis (PCA), metabolic profiles of the five-time point serum samples were markedly different, with the metabolome being changed over the course of nCRT (Fig. 1c). Next, we used the SAM method^29^ (Significance Analysis of Microarrays) to identify metabolites that associate with the nCRT treatment. A total of 219 metabolites were significantly changed over the course of nCRT (FDR adjusted *P* < 0.05; Supplementary Data 2). Among them, the quantities of 119 metabolites were increased and 100 metabolites were decreased over the course of nCRT (Fig. 1d). Metabolic pathway enrichment analysis revealed that the 219 altered metabolites were represented in amino acid metabolism including valine, leucine and isoleucine biosynthesis, arginine biosynthesis, and glycine, serine and threonine metabolism (Fig. 1e).

Further examination using hierarchical clustering analysis (HCA) showed that the nCRT induced alterations in metabolic dynamics were distinctive among the changed metabolites (Fig. 1f, g). For metabolites that were increased during nCRT, two different clusters were generated. Levels of metabolites in cluster 1 such as hypoxanthine and uracil were increased with the number of nCRT fractions, whereas the majority of metabolites in cluster 2 had little changes until the 25th nCRT. Similarly, two clusters (i.e., clusters 3 and 4) were observed for metabolites that were decreased over the course of nCRT. Levels of metabolites in cluster 3 were decreased significantly at the 15th nCRT and maintained the lower levels until surgery (Time 5). For examples, metabolites in tryptophan metabolism decreased significantly after nCRT (Supplementary Fig. 2). In contrast, metabolites in cluster 4 such as ornithine and citrulline decreased at the 15th nCRT, but before surgery, levels of these metabolites restored to the baseline (Time 1). Taken together, comprehensive metabolomics demonstrated that nCRT treatment induced significant changes in metabolic profiles and identified responsive metabolic traits over the course of nCRT in LARC patients.

### Acyl carnitines are linked to sex disparity in diarrhea induced by nCRT

**Fig. 2:**
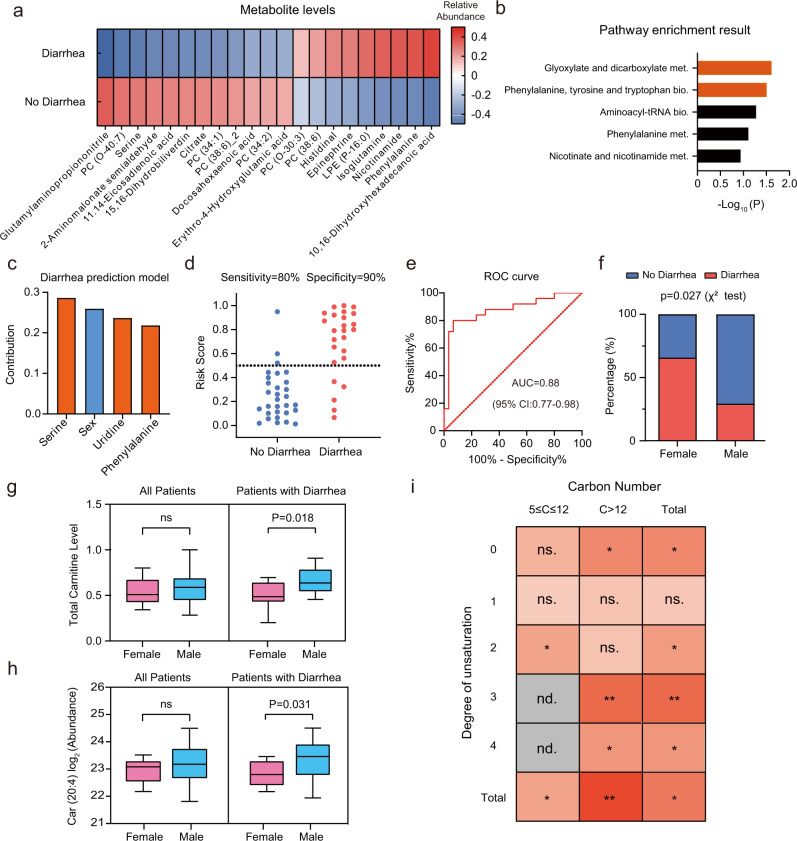
Acyl carnitines are linked to sex disparity in diarrhea induced by nCRT. **a** Pre-therapeutic metabolites in baseline were significantly associated with diarrhea of LARC patients (*P* < 0.05; Two-sided Wilcoxon test). All metabolites were corrected by the covariate of sex using a linear model. **b** Pathway enrichment analysis (*P* < 0.05; Hypergeometric test). **c** Selected key metabolites and the covariate of sex for the logistic regression model to predict the chance of diarrhea. **d** Sensitivity and specificity of the prediction model with the risk score of 0.5 (Diarrhea: *n* = 25; No diarrhea: *n* = 30). **e** Receiver operating characteristic curve of the logistic regression model. **f** Female patients had a significant higher chance of diarrhea than males (*P* < 0.05; *χ*^2^ test). **g**, **h** Levels of total carnitines and Car (20:4) between females and males in all patients (Female: *n* = 17; Male: *n* = 38) and patients with diarrhea (Female: *n* = 12; Male: *n* = 13). The centerline of boxes depicts the median values; the bottom and top box edges correspond to the first and third quartiles, and whiskers indicated the minimum and maximum values. **i** Sex disparity of carnitine levels related to diarrhea induced by nCRT (nd., not detected; **, *P* < 0.01; *, *P* < 0.05; ns., not significant; Two-sided Wilcoxon test).

Diarrhea is one of the most common adverse events induced by neoadjuvant chemoradiotherapy in locally advanced rectal cancer patients, which compromises the treatment efficacy and clinical outcomes^12,30^. To investigate metabolic traits associated with diarrhea induction, we analyzed the metabolomics data at baseline (Time 1) from patients with (*n* = 25) and without diarrhea (*n* = 30) before they received nCRT. Initial analysis identified 21 metabolites that were associated with diarrhea (*P* < 0.05; Wilcoxon test; Fig. 2a). Pathway analysis showed that these metabolites were significantly enriched in glyoxylate and dicarboxylate metabolism, and phenylalanine, tyrosine and tryptophan biosynthesis (*P* < 0.05; Hypergeometric test; Fig. 2b). Next, we used adaptive lasso regression to select key metabolites and constructed a logistic regression model to predict the chance of diarrhea in LARC patients before they received nCRT. Three metabolites, serine, uridine, phenylalanine, and the clinical covariate of sex were selected out for the prediction model (Fig. 2c and Supplementary Table 2). The cut-off score for prediction was 0.50 with the diagnostic sensitivity of 90% and the specificity of 80% (Fig. 2d). The receiver operating characteristic (ROC) curve also confirmed the good discriminative performance (AUC = 0.88; 95% CI: 0.77–0.98, Fig. 2e). Of note, this model enabled to predict LARC patients with all levels of diarrhea (Supplementary Fig. 3a). Based on the sample sizes of 25 cases and 30 controls, we found that all of four factors had sufficient powers for diarrhea prediction (powers of four factors in model larger than 0.8 with *α* = 0.05; Supplementary Table 3)^31^. These results showed that the pre-therapeutic metabolic traits are markedly different between patients with and without the nCRT induced diarrhea, and metabolite levels prior to therapy have potential for identifying individuals who are at the high risk of nCRT toxicity.

Previous studies have reported the sex-related differences in the nCRT induced adverse events such as diarrhea, with the female sex being a risk factor^15^. In our study, a significant higher incidence of the induced diarrhea was also observed in female patients than in male patients (*P* < 0.05; Chi-square test; Fig. 2f). Then, we sought to investigate the sex-specific differences in metabolic traits related to the nCRT induced diarrhea. Comparative analyses of metabolites between female and male patients with diarrhea revealed that 29 metabolites were associated with sex and diarrhea (*P* < 0.05; Wilcoxon test). Among them, 13 metabolites showed no differences between all female and male patients while the diarrhea factor was unstratified (Supplementary Fig. 3b), which suggests that these metabolites have sex-specificity in patients with diarrhea only. Closer examination revealed that three carnitines (i.e., Car (12:2), Car (13:0) and Car (20:4)) were significantly linked with the sex-specific differences in the nCRT induced diarrhea. Interestingly, total serum carnitine summed by 34 detected carnitines had a significantly higher level in male patients with diarrhea than that in female patients (Fig. 2g and Supplementary Data 3). As a comparison, this sex disparity in total carnitine level was not observed for all patients combined. For example, Car (20:4) has been found to display a higher level in males with diarrhea than that in females with diarrhea, while no difference was observed between the two sexes for all patients combined (Fig. 2h). The similar results for Car (12:2) and Car (13:0) were provided in Supplementary Fig. 3b. The comparison for the levels of total carnitine and individual carnitines among four groups (males with diarrhea, males without diarrhea, females with diarrhea, females without diarrhea) was showed in Supplementary Fig. 4. Furthermore, subsequent analyses of carnitines on the basis of carbon number and unsaturation degree revealed additional sex-related differences. We found that saturated long chain carnitines (C > 12) had significant differences between males and females with diarrhea, whereas saturated middle chain carnitines (5 ≤ C ≤ 12) showed no sex differences (Fig. 2i). Middle chain carnitines with two carbon-carbon bonds and long chain carnitines with high degree of unsaturation (≥3) held sex differences in the nCRT induced diarrhea. Combined, these results clearly showed that the sex-specific differences in metabolism is linked to the diarrhea induced by nCRT.

### Pre-therapeutic metabolic traits predict the nCRT induced hematologic toxicities

**Fig. 3:**
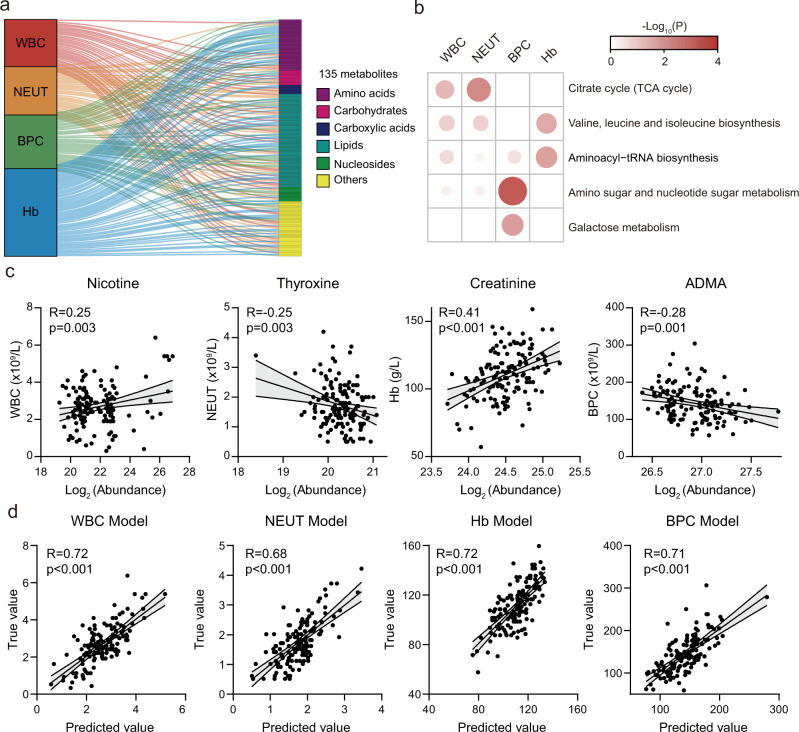
Pre-therapeutic metabolic traits predict the nCRT induced hematologic toxicities. **a** Significant correlations between pre-therapeutic serum metabolites (*n* = 135) and cell counts that indicate hematologic toxicity (Pearson correlation; two-sided Student’s *t* test *P* < 0.05). WBC, white blood cells; NEUT, neutrophils; BPC, blood platelet cells; Hb, hemoglobin. **b** Pathway enrichment analyses of hematologic toxicity associated metabolites (Hypergeometric test; *P* < 0.05). **c** Four metabolite examples and their correlations with hematologic toxicities. ADMA represents asymmetric dimethylarginine (Pearson correlation; Two-sided Student’s *t* test *P* < 0.05). Error bands represent 95% confidence intervals. **d** Multiple linear regression-based models using baseline metabolites in serum to predict lowest cell counts and hematologic toxicity during nCRT (Pearson correlation; Two-sided Student’s *t* test *P* < 0.05). Details of prediction models were provided in Supplementary Tables 4–7. Error bands represent 95% confidence intervals.

Hematologic toxicity frequently occurrs in locally advanced rectal cancer (LARC) patients who received neoadjuvant chemoradiotherapy (nCRT), which also contributes to cessation of treatment for patients who are unable to tolerate high dose of radio- and chemo- therapies, and reduces the nCRT therapeutic efficacy^13,14^. We next examined how the pre-therapeutic metabolite levels prior to nCRT (baseline; Time 1) are correlated with common hematologic toxicities, such as the lowest cell counts of white blood cells (WBC), neutrophils (NEUT), and blood platelet cells (BPC), and the lowest level of hemoglobin (Hb). These values were measured for individual patients during nCRT. We identified that 135 metabolites from different chemical classes had significant correlations with at least one of the hematologic toxicities (Pearson correlation; *P* < 0.05; Fig. 3a and Supplementary Data 4). Pathway enrichment analyses showed that the WBC, NEUT, and Hb correlated metabolites were significantly enriched in similar metabolic pathways, such as citrate cycle and aminoacyl-tRNA biosynthesis (Fig. 3b). As a comparison, BPC correlated metabolites were highly enriched in galactose metabolism and amino sugar and nucleotide sugar metabolism (Fig. 3b). As shown in Fig. 3c, we demonstrated additional metabolite examples including nicotine, thyroxine, creatinine, and asymmetric dimethylarginine (ADMA) and their correlations with hematologic toxicities.

Since all metabolites were measured at baseline prior to nCRT, we next sought to predict hematologic toxicities using the dysregulated metabolites for LARC patients. Similar to the diarrhea prediction, we first selected key metabolites and constructed the prediction models using multiple linear regression (MLR) models. As a result, 15, 12, 12, and 17 metabolites were selected, respectively, for the prediction models of WBC, NEUT, Hb, and BPC values (Fig. 3d and Supplementary Tables 4–7). Each of the generated prediction models successfully predicted the WBC, NEUT, Hb, and BPC values using the baseline metabolite levels before patients received nCRT, and had significant and positive correlations with the clinically measured values during nCRT treatment (Fig. 3d). Altogether, we demonstrated that pre-therapeutic metabolic traits in the serum of LARC patients could successfully predict the chances of various hematologic toxicities during nCRT, which further indicated that serum metabolic profiles of LARC patients are key determinates of the nCRT induced toxic events.

### Phenylalanine metabolism underlies distinct therapeutic responses of nCRT

**Fig. 4:**
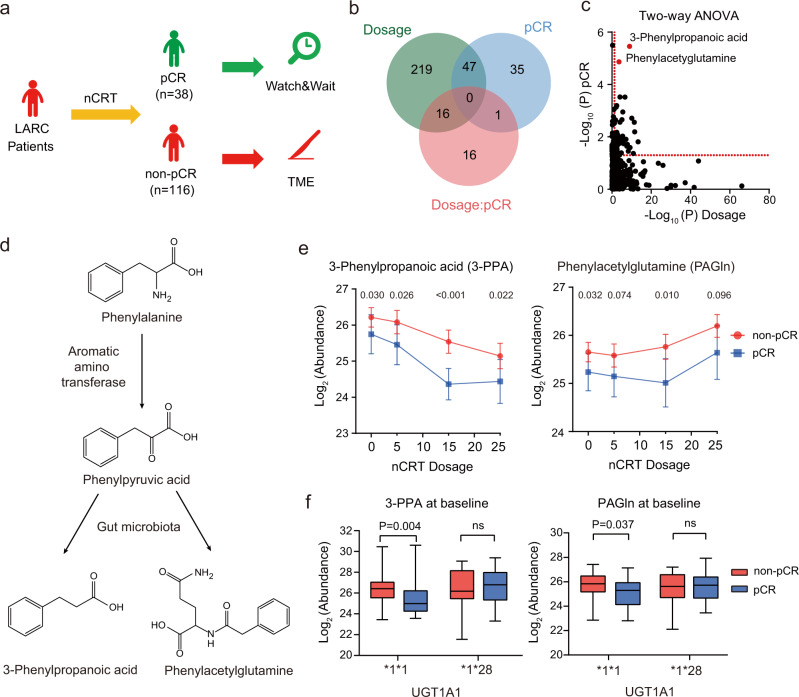
Phenylalanine metabolism underlies distinct therapeutic responses of nCRT. **a** Different therapeutic strategies for pCR and non-pCR patients. **b** Differentially changed metabolites related to nCRT dosage, pCR status, and both combined using the two-way ANOVA analysis (*P* < 0.05). **c** 3-Phenylpropanoic acid and phenylacetylglutamine are the most significant metabolites related to nCRT dosage and pCR status. The red dash line represents the cut-off of P value 0.05 (Two-way ANOVA test). **d** The scheme for the metabolic pathway of 3-phenylpropanoic acid and phenylacetylglutamine. **e** Relative abundances of 3-phenylpropanoic acid and phenylacetylglutamine in response to nCRT dosage (At individual time points, *n* = 38, 35, 37, 38 for pCR and *n* = 116, 96, 113, 116 for non-pCR). Two-sided Wilcoxon test. The dot depicts the mean values. Error bars represent 95% confidence intervals. **f** Levels of 3-phenylpropanoic acid and phenylacetylglutamine between pCR and non-pCR patients in different *UGT1A1* genotypes (*1*1: pCR, *n* = 21, non-pCR, *n* = 73; *1*28: pCR, *n* = 12, non-pCR, *n* = 18). Two-sided Wilcoxon test; ns, not significant. The centerline of boxes depicts the median values; the bottom and top box edges correspond to the first and third quartiles, and whiskers indicated the minimum and maximum values.

With the completion of neoadjuvant chemoradiotherapy, approximately 10% to 35% of patients achieve pathological complete response (pCR) and could be selected for the watch-and-wait strategy while the non-pCR patients are guided to the TME surgery immediately^32^. To investigate the metabolic alterations reflective of therapeutic responses over the course of nCRT, we analyzed the metabolic profiles at baseline (Time 1) and after 25th nCRT (Time 4) for pCR (*n* = 38) and non-pCR (*n* = 116) patients (Fig. 4a). A two-way ANOVA analysis was used, and 282 and 83 metabolites were identified as in relation to nCRT dosage and pCR status, respectively (Fig. 4b and Supplementary Data 5). Notably, two metabolites in phenylalanine metabolism, 3-phenylpropanoic acid (3-PPA) and phenylacetylglutamine (PAGln), were found to have the most prominent changes between pCR and non-pCR patients (Fig. 4c). Phenylalanine is first metabolized to phenylpyruvic acid by aromatic amino transferase, then converted to 3-PPA and PAGln with presence of gut microbiota^33,34^ (Fig. 4d and Supplementary Fig. 5). Our analyses showed that 3-PPA was significantly higher in non-pCR patients at all time points (Fig. 4e). PAGln also showed a higher level in non-pCR patients, but at baseline and after 15th nCRT only. There was also a trend of difference between patients with pCR and non-pCR after 5th nCRT (*P* = 0.074) and after 25th nCRT (P = 0.096). The level of 3-PPA was decreased gradually with nCRT dosages in non-pCR patients. In contrast, no further reduction of PAGln was observed in pCR patients after the 15th nCRT. Before surgery, the significant difference in 3-PPA was still kept between pCR and non-pCR patients (Supplementary Fig. 6a). We checked the metabolomics data from another independent cohort from one of our previous publications^35^, and verified that pheylacetylglutamine (PAGIn) is also closely associated with the status of colon rectal cancer. The results demonstrated that levels of PAGIn were significantly higher in plasma samples of CRC patients compared to those in polyp controls. Similarly, for CRC patients, levels of PAGIn were also significantly deceased after tumor removal surgery (Supplementary Fig. 7).

We also checked the association between the two metabolites and the toxicity of the treatment and the hematologic toxicities of the patients at baseline. Pre-therapeutic serum levels of the two metabolites between patients with diarrhea and without diarrhea showed no differences in 3-PPA and PAGln between the two groups (Supplementary Fig. 8a, b). Correlation analysis showed that 3-PPA had a weak negative association with white blood cells (WBC) (*P* < 0.05, *r* = −0.18) and PAGln also presented a weak negative association with the lowest level of hemoglobin (Hb) (*P* < 0.05, *r* = −0.18; Supplementary Fig. 8c).

To address the association between gut bacteria involved in phenylalanine metabolism and therapeutic responses of nCRT, we further analyzed the microbiome data at baseline in the previous independent cohort in which none of the patients overlapped with our study^36^. According to the drug treatment, pCR patients (*n* = 26) and non-pCR patients (*n* = 46) treated with the combination of capecitabine with irinotecan were selected for analysis using the Linear Discriminant Analysis (LDA). As the results showed in Supplementary Fig. 9, 14 taxa were significantly different between pCR and non-PCR patients (*P* < 0.05, LDA score > 2). In particular, families in Clostridiales were showed to have lower levels in pCR patients compared with that in non-pCR patients. For example, *Clostridium IV*, which has been reported to have a positive association of 3-phenylpropanoic acid (3-PPA) production^37^, was found to have a significant lower level in pCR patients compared with that in non-pCR patients (Supplementary Fig. 9c). Given that the read for species *Clostridium sporogenes* was not detected, we analyzed the levels of genus and identified a notable trend of lower levels of *Clostridium sensu stricto* in pCR patients compared with those in non-pCR patients albeit that the P value was calculated as 0.07 (Supplementary Fig. 9d). Overall, we showed that in an independent cohort, gut microbiota that associated with aromatic amino acid metabolism were linked to the pCR status of patients treated with nCRT for colorectal cancer.

Our previous phase III clinical trial has demonstrated that nCRT guided by *UGT1A1* genotyping significantly improved the pCR rate of LARC patients from 15% to 30%^27^. Therefore, we examined the pre-therapeutic differences in 3-PPA and PAGln between pCR and non-pCR patients on the basis of *UGT1A1* genotyping. Interestingly, patients with UGT1A1*1*1 genotype showed significantly higher levels of 3-PPA and PAGln in non-pCR group than those in pCR group in baseline serum samples prior to nCRT (Fig. 4f). However, elevations of two metabolites were not observed in patients with UGT1A1*1*28 genotype. The significant difference in 3-PPA was still maintained between pCR and non-pCR groups in patients with UGT1A1*1*1 genotype after 15^th^ nCRT (Supplementary Fig. 6b). Therefore, the results suggest that both *UGT1A1* genotype status and serum metabolite levels contribute to the therapeutic responses of nCRT. For patients with UGT1A1*1*1 genotype, the lower serum levels of 3-PPA and PAGln indicate a higher chance for pCR. The genetic and metabolic factors are interplayed and closely associated with the nCRT efficacy. These findings highlighted the importance of phenylalanine metabolism underlying the therapeutic responses of the *UGT1A1* genotyping-based nCRT.

### Elevated essential amino acids are beneficial to pCR in nCRT

**Fig. 5:**
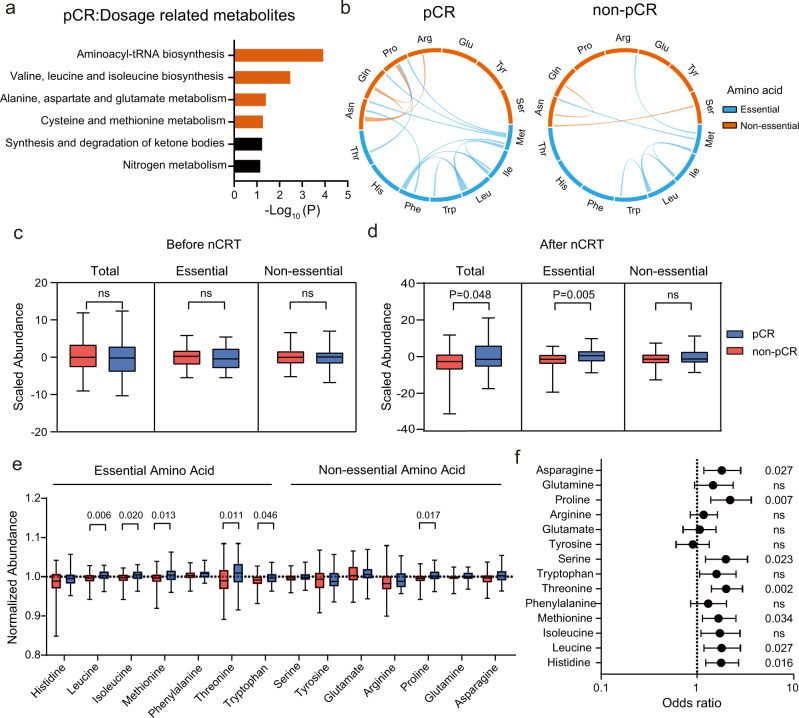
Elevated essential amino acids are beneficial to pCR in nCRT. **a** Pathway enrichment analysis of metabolites related to both nCRT dosage and pCR status (Hypergeometric test; *P* < 0.05). **b** Metabolic correlation between two amino acids in pCR and non-pCR patients (Pearson correlation; *r* > 0.60 and two-sided Student’s *t* test *P* < 0.05). **c**, **d** Levels of amino acids in serum samples of pCR (*n* = 38) and non-pCR (*n* = 116) patients before and after nCRT: **c**, before nCRT at baseline; d, after the 25th nCRT (Two-sided Wilcoxon test; ns, not significant). **e** Levels of amino acids in serum samples between pCR (*n* = 38) and non-pCR (*n* = 116) patients after nCRT. The data was normalized to the levels in baseline (Two-sided Wilcoxon test). The centerline of boxes depicts the median values; the bottom and top box edges correspond to the first and third quartiles, and whiskers indicated the minimum and maximum values. **f** Odd ratios of amino acids after nCRT related to the pCR status (two-sided z-test of logistic regression; ns, not significant). pCR (*n* = 38) and non-pCR (*n* = 116). Error bars represent 95% confidence intervals.

In the two-way ANOVA analysis, we also found that 33 metabolites had significant interactive effects on both neoadjuvant chemoradiotherapy (nCRT) dosage and pathological complete response (pCR) status (within red circle; Fig. 4b). Pathway enrichment analysis of these metabolites showed that the amino acid related pathways were significantly enriched (Fig. 5a). We next investigated the metabolic coordination between amino acids before and after the nCRT treatment (Times 1 and 4) in pCR and non-pCR patients. Pearson correlation analyses identified 15 positively correlated amino acids in pCR patients, with up to 11 of them being present for essential amino acids. Nevertheless, only 7 positive correlations were found in non-pCR patients (Fig. 5b, Supplementary Fig. 10, and Supplementary Data 6). Closer examinations of the total, essential, and non-essential amino acids showed no differences between pCR and non-pCR patients at baseline prior to nCRT (Fig. 5c). Strikingly, after the completion of nCRT (Time 4, after the 25th nCRT), significantly higher levels of essential and total amino acid levels in pCR patients than that in non-pCR patients were observed. As a comparison, no differences were found in the levels of non-essential amino acids (Fig. 5d). Comparative analyses of individual amino acids showed that pCR patients had higher levels of major essential amino acids including leucine, isoleucine, methionine, threonine, and tryptophan that those in non-pCR patients (Fig. 5e). For non-essential amino acids, only proline showed significant difference between pCR and non-pCR patients. Next, we constructed a logistic regression model and calculated the odds ratio for each amino acid, and investigated the contribution of amino acid to pCR status (Fig. 5f). The results showed that asparagine, proline, serine, threonine, methionine, leucine, and histidine had odds ratios higher than 1, suggesting that higher levels of these amino acids are beneficial to pathological complete response with nCRT treatment. Collectively, the results demonstrated that amino acids are significant metabolic traits to reflect both nCRT dosage and pCR status for LARC patients. In particular, essential amino acids had no differences at baseline prior to nCRT but were significantly elevated in the serum samples of pCR patients after the completion of nCRT. The data also suggests that diet and nutrition intervention especially supplementation of essential amino acids during nCRT may have a beneficial contribution to tumor remission for LARC patients.

## Discussion

In this study, we characterized the serum metabolic profiles of locally advanced rectal cancer patients with neoadjuvant chemoradiotherapy and identified responsive metabolic traits associated with the treatment course, toxicities, and therapeutic responses. Our initial analyses comparing five time points revealed marked differences in metabolic responses over the course of nCRT. Amino acid metabolism pathways including valine leucine and isoleucine biosynthesis, arginine biosynthesis, and glycine serine and threonine metabolism were significantly affected in response to nCRT treatment. We found that metabolites in tryptophan metabolism such as tryptophan, kynurenic acid, indole-3-acetic acid, and indole-3-lactic acid were exclusively decreased at the 25th nCRT (Supplementary Fig. 2). A previous study by Guo et al. showed that two tryptophan metabolites, kynurenic acid and indole-3-carboxaldehyde, have a long-term radioprotective function in a mouse model^23^. Wang et al. also reported the up-regulation of tryptophan metabolism as a result of irinotecan-induced intestinal damage in mouse gut^24^. On the other hand, cancer cells have high levels of tryptophan metabolites to enhance tumor malignancy through aryl hydrogen receptor (AHR) activation^38^. Given its roles in both regulating tumor growth and inducing chemoradiotherapeutic effects, the decreased tryptophan metabolites discovered in our LARC patient cohort may account for this interrelation during nCRT.

Chemoradiotherapy is often accompanied with adverse effects including diarrhea, leukopenia, and neutropenia, which contribute to unfavorable clinical outcomes of nCRT treatment^12–14^. Although studies have indicated the associations between endogenous metabolites and diarrhea^24,39^, our results found that the pre-therapeutic metabolic traits are considerably different between patients with and without diarrhea. More importantly, we reported a predictive model using the pre-therapeutic serum metabolites for diarrhea prediction in the context of nCRT. The combination of serine, uridine, phenylalanine and patient sex enabled differentiation between patients with and without diarrhea. We also established the relationships between metabolite levels at baseline and important hematologic toxicities, such as WBC, NEUT, Hb, and BPC values. We found that WBC, NEUT, and Hb correlated metabolites were enriched in TCA and aminoacyl-tRNA biosynthesis pathways, while BPC correlated metabolites were highly enriched in carbohydrate metabolism. These results highlighted the potential of serum metabolomic traits prior to therapy for identifying individuals who are at high risk of nCRT toxicity, which could enable individualized treatment for LARC patients.

A notable finding of this study is the sex-specific difference in carnitine metabolism that is linked to diarrhea induced by nCRT. Notably, a growing number of independent clinical trial studies also demonstrated that sex is a significant factor responsible for the disparity of adjuvant treatment-induced toxicity for CRC, with females being consistently at higher risk of diarrhea^15,40–43^. This agreement of sex factor identification between our prediction models and other independent studies strengthens the presented results to a certain extent. In our study, we revealed that female patients with diarrhea had significant lower levels of serum carnitines than that in male patients with diarrhea. Acyl carnitines are indispensable metabolite hubs for β-oxidation of fatty acids, which supply source of energy for cell growth and proliferation^44^. Sex hormones including estradiol and estrogen were showed to inhibit β-oxidation through action on carnitine palmitoyltransferase^45,46^. Although high dose of L-carnitine administration has been reported to cause diarrhea in healthy individuals^47^, it is unclear the intertwined links between carnitine metabolism mediated by sex hormones and the diarrhea induced during chemoradiotherapy. Our results suggest another potential nutrient intervention avenue for female patients with diarrhea in the context of nCRT, and further experimental and clinical studies are warranted.

With treatment of nCRT, only 10–35% of LARC patients can achieve pathological complete response. Since the status of pathological complete response cannot be confirmed before surgery, presurgical examination of pCR with high accuracy is invaluable in guiding the selection of patients appropriate for the watch-and-wait strategy^7^. We found that pCR patients had significantly lower levels of 3-phenylpropanoic acid (3-PPA) and phenylacetylglutamine (PAGln) than non-pCR patients. It has been reported that phenylacetylglutamine was significantly increased in serum and urine of CRC patients^48,49^, indicating that PAGln is possibly linked to colorectal cancer progression. 3-phenylpropanoic acid is metabolized through reductive pathway of phenylalanine metabolism by the gut symbiont *Clostridium sporogenes*^50^. A previous study demonstrated that genetic modulation of *Clostridium sporogenes* metabolic products, aromatic amino acids, alters host intestinal permeability and immune activation^33^. In addition, nCRT responders and non-responders differ remarkably in gut microbiome components^36^. A previous study by Colosimo et al. showed that bacterial derived 3-PPA can interact with G protein-coupled receptors (GPCRs) associated with diverse functions within the nervous and immune systems, among others^51^. Nemet et al. also reported that PAGln can mediate cellular responses via GPCRs and act via adrenergic receptors (ADRs)^52^. Though there are limited functional studies on 3-PPA and PAGln, it has been suggested those aromatic amino acids participate in the interplay between gut microbes and host immune response through interactions with key signaling receptors such as GPCRs. GPCRs are signaling receptors that function in many cancers by regulating cellular proliferation, invasion, migration, immune cell-mediated functions, angiogenesis and survival at metastatic sites^53^. Proliferation of many cancers is stimulated by GPCR agonists^54^. Therefore, we speculate that the interplay between the two metabolite abundances and the signaling pathways is a potential determinant for response to nCRT treatment. Our present study showed that the 3-PPA and PAGln, which were demonstrated as potential GPCR agonists, are risk factors for failure of achieving pathological complete response. The detailed mechanism of those metabolites may be related to the complex signaling affected by cancer and chemoradiation, which are strongly dependent on dose and individual heterogeneity, which requires further biological validation. Though the levels of phenylalanine in serum showed no significant differences between pCR and non-pCR, the two products of phenylalanine metabolism, 3-PPA and PAGln, which has been proved associated with gut microbiome^33,55^, showed significant differences between pCR and non-pCR patients, suggesting that the gut bacteria involved the different responses of nCRT therapy. Thus, we reasoned that gut bacterial metabolites, in particular the intermediates of phenylalanine metabolism, have potential in predicting therapeutic responses for LARC patients with nCRT treatment. Beyond that, we found that the differences in 3-phenylpropanoic acid and phenylacetylglutamine between pCR and non-pCR patients possessed specificity in *UGT1A1* genotype, wherein differences were seen in patients with UGT1A1*1*1 genotype but not in patients with UGT1A1*1*28 genotype. In irinotecan plus capecitabine-based nCRT, *UGT1A1* is responsible for inactivation of the SN-38, which is a metabolic product of irinotecan^16^. Given that the maximum tolerated dose of irinotecan decreases with an increase in the number of defective *UGT1A1* alleles, patients with UGT1A1*1*28 genotype are prone to have higher chance of the nCRT induced toxicities^27,56^. Therefore, it seems plausible that differences in 3-phenylpropanoic acid and phenylacetylglutamine seen in patients with UGT1A1*1*1 genotype could account for toxicity tolerance between pCR and non-pCR patients during the course of nCRT. This is also indicated by the result that pCR patients have enhanced essential amino acid metabolism. These results suggest that diet and nutrition intervention with supplementation of essential amino acids may have a positive contribution to improve responses to nCRT for non-pCR patients, but more clinical trials are required before this intervention can be implemented in clinical practice.

Previous studies have demonstrated the application potential of omics analysis for nCRT treatment in rectal and other cancers^25,26,36,57–60^ (Supplementary Table 8). For example, Rodriguez et al. identified that patients with pCR had lower level of valine at baseline and those with relapse had lower level of succinate by GC-MS based targeted metabolomics^26^. Diaz et al. performed metabolomics analysis in breast cancer patients with nCRT and found that glycohyocholic acid and glycodeoxycholic acid can classify triple-negative patients regarding treatment response^59^. Wang et al. identified numerous differentially expressed genes and miRNAs from microarray datasets of nCRT responder group in patients with esophageal squamous cell carcinoma^60^. However, the longitudinal analysis for the whole processing of nCRT is rare in study design and sample size of previous studies is small. Our previous work also identified 15 potential metabolite biomarkers to predict tumor response to neoadjuvant chemo-radiation therapy at baseline in patients with locally advanced rectal cancer^25^. Compared to previous studies, the present study is conceptually and technically different in terms of the following aspects: (1) Based on our results of a recent phase III study (CliClare), we enrolled patients (*n* = 165) with treatment of both capecitabine and irinotecan in the present study. (2) The cohort in the current study is a longitudinal study designed for longitudinal analyses, which can provide valuable insights into the dynamic metabolic traits in response to nCRT in patients with LARC. (3) The clinical endpoint in this study is the presence of pathological complete response (pCR), which is optimal and more stringent for clinical assessment than tumor regression grade (TRG) that was used in the previous study.

There are some limitations in the present study. The purpose of this study was not to develop a clinical test for prediction of nCRT responses, for which absolute concentrations of metabolites would be required, but to identify potential metabolites that could be associated with nCRT treatment course, toxicities, and therapeutic responses. Therefore, a targeted quantitative measurement method was not used for our study. However, it was feasible to use the peak areas of metabolite ions to compare levels of these metabolites and correlate them with the nCRT treatment. Although the sample size of our study is larger than many previous metabolomics studies on chemo- and radio-therapies, the small sample size is still a potential limitation for this study and further analysis in a larger cohort is required. We also hope to recruit an additional cohort of patients with samples from multiple sites for validation in the near future. To partially validate our results, we first analyzed another metabolomics study for colorectal cancer patients, which revealed that the PAGln was also linked to colorectal cancer progression (Supplementary Fig. 7). Most importantly, for CRC patients, levels of PAGIn were significantly deceased after tumor removal surgery. In addition, results from gut microbiota validated our findings that aromatic amino acid metabolism was correlated with the pCR status for LARC patients treated with nCRT (Supplementary Fig. 9). Besides, pathway enrichment analysis in this study was based on differential metabolites in the circulating metabolome, where actually pathways do not exist in contrast to tissue or cellular metabolomes. We do think the ideal experiment design should include tissue samples to identify metabolic pathways that are originally impacted by the nCRT treatment. However, it is challenging to obtain tissue samples from the patients over the treatment course of nCRT. Given that the tumor-originating metabolites enter the circulatory system, we speculate that the significantly changed metabolites detected in blood could reflect changes pertaining to tissue or cellular metabolome. In our previous publication, we analyzed dysregulated metabolites from paired tissue and plasma samples from the same CRC patients, and demonstrated that tissue-correlated metabolites in plasma accurately reflected the pathological status and tumor stages of CRC patients and have a high diagnostic potential for clinical applications^35^. In addition, metabolites produced by certain bacteria and dietary metabolism can enter the circulatory system. For example, glyoxylate and dicarboxylate metabolism was enriched because of the dysregulated changes in serine and citrate. Although there is an ongoing debate what pathway analysis is suitable to analyse the circulating metabolome, many studies have used the pathway enrichment analysis on the basis of circulating metabolome and revealed valuable insights into human health and diseases^61,62^. Though not the prominent focus in the present study, we believe that the pathway analysis helps to interpret results in light of the whole-body metabolism which has been impacted by nCRT. Taken together, our primary results revealed therapeutic toxicities and responses of nCRT and showed potential benefits for LARC patients.

## Methods

### Sample collection

The study protocol was approved by the central ethics committee of Fudan University Shanghai Cancer Center (Shanghai, China). The serum samples (*n* = 743) of 165 LARC patients were collected from Fudan University Shanghai Cancer Center between July 2014 and January 2018. Patients were recruited in an experiment group of a multicenter, randomized, open-label phase III clinical trial in China (CinClare, ClinicalTrials.gov identifier: NCT02605265). Details of the cohort have been provided in the clinical report^27^. In brief, eligible patients were aged 18–75 years old and diagnosed with clinical stage T3-4 and/or N + rectal adenocarcinoma. The inclusion criteria included a Karnofsky performance status score ≥70, a *UGT1A1* genotype of *1*1 or *1*28, adequate bone marrow function (a hemoglobin level ≥9 g/dL, neutrophil count ≥ 1500/mL, and platelet count ≥100,000/mL), liver function (total bilirubin level <1.5 times the upper limit of normal; albumin level >30 g/L; and aspartate aminotransferase, alanine aminotransferase, and alkaline phosphatase levels <2.5 times the upper limit of normal), and normal kidney function (creatinine concentration below the upper limit of normal). The sex information of patients enrolled in this study was determined by self-reporting. Detailed information on sex is provided in Supplementary Table 1. All enrolled patients have provided informed consent. Patients were not compensated for their participation in the trial, but were provided with treatment free of charge. Patients received the complete treatment course of nCRT (50 Gy/25 fractions; concurrent capecitabine + irinotecan chemotherapy) and 1 cycle of interval chemotherapy (CAPIRI, capecitabine+ irinotecan). The dosage of irinotecan was determined by *UGT1A1* genotype. For patients with the UGT1A1*1*1 genotype and UGT1A1*1*28 genotype, the weekly doses of irinotecan were administered at 80 mg/m^2^ and 65 mg/m^2^, respectively. In this study, diarrhea diagnosis was determined by clinical symptoms according to CTCAE (Common terminology criteria for adverse events, version 4.0). In brief, patients with an increase of <4 stools per day over baseline and mild increase in ostomy output compared to baseline were diagnosed as grade 1 diarrhea; patients with an increase of 4–6 stools per day over baseline and moderate increase in ostomy output compared to baseline were diagnosed as grade 2 diarrhea; patients with an increase of ≥7 stools per day over baseline, incontinence, hospitalization indicated and severe increase in ostomy output compared to baseline were diagnosed as grade 3 diarrhea; patients with diarrhea induced life-threatening consequences and even death were diagnosed as grade 4 and grade 5 diarrhea. Sex was also considered in analysis as it is known that females had higher incidence of the nCRT induced diarrhea than males. In this study, no grade 4 or grade 5 diarrhea occurred during nCRT. In this study, pCR was defined as pathological T0N0M0 and all pCR statuses were evaluated by two independent pathologists. If their conclusions were inconsistent, it was evaluated again by a third pathologist. Serum samples for each patient were collected at the following time points: before nCRT (Time 1), at the 5th fractions of nCRT (Time 2), at the 15th fractions of nCRT (Time 3), at the 25th fractions of nCRT (Time 4), and after the rest for two months and within 2 days before surgery (Time 5) (Supplementary Fig. 1). For serum collection, all participants were in an overnight fasting state, and 5 mL of peripheral venous blood was drawn in the morning.

### Reagents and sample preparation

LC−MS grade water (H_2_O) and methanol (MeOH) were purchased from Honeywell (Muskegon, USA). Ammonium hydroxide (NH_4_OH) and ammonium acetate (NH_4_OAc) were purchased from Sigma-Aldrich (St. Louis, USA). Chemical standards of metabolites were purchased from J&K (Beijing, China), Sigma (St. Louis, USA), Carbosynth (Berkshire, UK), TCI (Tokyo, Japan), and Energy Chemical (Shanghai, China). Serum samples (50 µL) were extracted using 150 μL MeOH with internal standards (d3-leucine and d8-phenylalaine). The samples were then vortexed for 30 s and sonicated for 15 min. To precipitate proteins, the samples were incubated for 1 h at −20 °C, followed by 15 min centrifugation at 17,500×*g* and 4 °C. The supernatants were transferred to HPLC vials and stored at −80 °C prior to LC−MS analysis.

### LC−MS analysis

The LC−MS analysis protocol followed our previous publication^63^. The data acquisition was performed using a Vanquish UHPLC coupled to a Orbitrap Exploris 480 (ThermoFisher Scientific, United States). The raw data was acquired using Xcalibur (version 4.4.16.14). A Waters ACQUITY UPLC BEH amide column (particle size, 1.7 μm; 100 mm (length) × 2.1 mm (i.d.)) and UPLC HSS T3 column (1.8 μm; 100 mm (length) × 2.1 mm (i.d.)) was used for the LC separation and the column temperature was kept at 25 °C. For amide column, mobile phase A was water with 25 mM ammonium hydroxide (NH_4_OH) and 25 mM ammonium acetate (NH_4_OAc), and B was ACN for both the positive (ESI+) and negative (ESI−) modes. The flow rate was 0.5 mL/min and the gradient was set as follows: 0–0.5 min, 95% B; 0.5–7 min, 95% B to 65% B; 7–8 min, 65% B to 40% B; 8–9 min, 40% B; 9–9.1 min, 40% B to 95% B; 9.1–12 min, 95% B. The injection volume was 2 μL. For T3 column, mobile phase A was water with 0.1% formic acid, and B was ACN with 0.1% formic acid for both the positive (ESI+) and negative (ESI−) modes. The flow rate was 0.5 mL/min and the gradient was set as follows: 0–8 min: 1% B to 99%B; 8–10 min: 99% B; 10–10.1 min, 99% B to 1% B; 10.1–12 min: 1% B; The injection volume was 2 μL. All the samples were randomly analyzed during data acquisition. The QC sample was prepared by pooling aliquots of all subject samples and injected every 20 samples.

The data acquisition was operated in full MS scan mode and dd-MS2 scan mode. The source parameters were set as follows: spray voltage, 3500 V or −2800 V for positive or negative mode, respectively; aux gas heater temperature, 350 °C; sheath gas, 50 arb; aux gas, 15 arb; capillary temperature, 400 °C. The resolution for full MS scan mode was set as 60,000 and AGC target was set as 1e6 for both positive and negative modes. Maximum IT was set as 100 ms. Mass range was set as 70–1200 Da. For the dd-MS2 scan mode, MS resolution was set as 30,000 and AGC target was set at 1e5. Maximum IT was set as 60 ms. The Top N setting was set as 6. Isolation width was set as 1.0. The collision energy was set as SNCE 20-30-40%. The dynamic exclusion was set as 3.0 s and isotope exclusion was on.

### Metabolomics data processing

The metabolomics data processing protocol followed our previous publication^63^. ProteoWizard (version 3.0.20360)^64^ was used to convert raw MS data (.raw) files to the mzXML format, and R package “XCMS” (version 3.12)^65^ was used for peak detection, retention time correction, and peak alignment. The XCMS processing parameters were set as follows: mass accuracy for peak detection = 10 ppm; peak width c = (5, 30); snthresh = 3; minfrac = 0.5. For each metabolic feature, the intensity more than 5 SD were considered as outlier and set as a missing value. The features with more than 70% of missing values in QC samples was removed. The remaining missing values were imputed using the k-Nearest Neighbor (KNN) algorithm^66^. The generated peak table was uploaded to MetFlow (http://metflow.zhulab.cn/)^67^ for normalization and integration to remove unwanted systematic errors based on QC samples. Metabolic peaks with RSDs less than 30% in QC samples were used for subsequent analysis. Metabolite identification was performed using our previously published software MetDNA (http://metdna.zhulab.cn/)^63^. In brief, we used an in-house metabolite spectral library for metabolite annotation by matching accurate mass, retention time and MS/MS similarity. The matched metabolites were considered as level 1 identification according to MSI^28^. Metabolite identifications with MSI level 2 confidence were achieved by matching accurate mass and MS/MS similarity. External public metabolite library and lipid spectral library were used. The rest of the metabolite identifications annotated from MetDNA were considered as MSI level 3. The MS/MS spectral similarity was calculated using the dot-product algorithm and the cutoff was set as 0.8. All metabolite and lipid identifications were provided in Supplementary Data 1. Two internal standards were spiked into individual samples (d3-leucine and d8-phenylalanine) to monitor the reproducibility during the LC−MS data acquisition, with the relative standard deviations (RSDs) of peak areas calculated as 3.2% and 4.7%, respectively (Supplementary Fig. 11a). After data normalization, the median RSDs of metabolites measured in HILIC − MS and RPLC−MS were 11.4% and 12.0% (Supplementary Fig. 11b), respectively. Principal components analysis (PCA) was conducted to assess the reproducibility of QC samples (Supplementary Fig. 11c). QC samples clustered tightly in PCA plot for both HILIC and RPLC modes. Those results indicated the excellent reproducibility and good data quality.

### Microbiome data processing

The gut microbiome dataset was from an independent cohort which was previous published^36^. The processed OTU abundance table and taxonomic annotation of the study was used for further validation. Paired-end raw sequences were merged using FLASH (version 1.2.8) and clean sequences were obtained after quality checking with fqtrim (version 0.9.4). The removal of chimeras, generation of representative sequences and operation taxonomy units (OTU) feature table were completed by Vsearch (version 2.11.1). The representative sequences were aligned to the Ribosomal Database Project classifier for taxonomic annotation. Only patients received combination therapy of irinotecan and capecitabine was selected. Among them, 46 patients were non-pCR while 26 were pCR. The features without more than 3 counts in 10% of samples were removed. The data was normalized by total sum scaling (TSS). The Linear Discriminant Analysis (LDA) effect size (LEfSe) were performed by “MASS” (v7.3.54) package of R. The R packages “phyloseq” (version 1.38.0) and “ggtree” (version 3.2.1) were used for visualization.

### Statistical analyses

The statistical analyses were performed using R (version 3.6.1). The SAM (Significance Analysis of Microarrays)^29^ was performed using an R package “samr” to identify the metabolites altered during nCRT treatment course. The distribution-independent ranking tests (based on the Wilcoxon test) and the sample-wise permutation were used to ascertain significance (false discovery rate, FDR < 0.05).

The pathway enrichment analysis was performed using MetaboAnalyst (https://www.metaboanalyst.ca/)^68^ embedded with the Kyoto Encyclopedia of Genes and Genomes (KEGG) pathway database^69^ (accessed on October, 2019). Hypergeometric test was used to calculate *P* value and out-degree centrality for topology analysis. All compounds in the selected pathway library was used for reference metabolome. Though we selected homo sapiens as the species for database search, some enriched pathways were mixed with mammalian and non-mammalian pathways, due to a long-standing and unresolved pathway curation issue in KEGG database. The detailed information of enriched metabolic pathways was provided in Supplementary Data 7.

The R package “pheatmap” (version 1.0.12) was used for hierarchical clustering analysis (HCA) and the median level was used to represent the level of all patients. The R package “glmnet” (version 4.0.2) was used for feature selection by the adaptive lasso^70^. At first, all metabolites and clinical covariates (age, sex, and BMI) were scaled and used in the selection of predictors. The adaptive weights vector **ω** is calculated as:

1 $${{{{{\boldsymbol{\omega }}}}}}=\frac{1}{{\left(\left|{{{{{{\boldsymbol{\beta }}}}}}}^{{ini}}\right|\right)}^{\gamma }}$$

Where ***β***^*ini*^ is the initial estimate of the coefficients obtained through ridge regression with a 10-fold cross-validation. *γ* is a positive constant for adjustment of the adaptive weights vector. In this study, it was set as 1. Subsequently, the adaptive weights vector **ω** was applied in the lasso regression as penalty factor. The optimal lambda was chosen by a 10-fold cross-validation. After the feature selection, the logistic regression was used to build the model for predicting the chance of diarrhea using serum metabolites at the baseline. Additionally, to consider the limited sample size, we restricted the number of predictors no more than four in the final model. The logistic model using four predictors to predict the chance of diarrhea is finally expressed as:

2 $${Risk\; Score}=\frac{{{{{{{\rm{e}}}}}}}^{{Logit}\left(P\right)}}{1+{{{{{{\rm{e}}}}}}}^{{Logit}\left(P\right)}}$$

3 $${Logit}\left(P\right)	=-0.14-1.07\times {M}_{{Uridine}}-1.30\times {M}_{{Serine}}\\ 	\quad+ 0.98\times {M}_{{Phenylalaine}}+1.17\times {Sex}$$

The logistic model was tested by bootstrapping using the R package “bool” (Supplementary Fig. 12). In brief, 63% randomly selected patients from the dataset were selected as discovery data to build the prediction model, and the remaining 37% patients were used as validation data. This random sampling with model construction and validation procedure was repeated 1000 times. Powers of predictors in diarrhea prediction model (Supplementary Table 3) were calculated by the G*Power software (version 3.1.9.4) and a z-test with two tails specific for the logistic regression was used.

The similar feature selection method was used in the predictions of hematologic toxicity. In this prediction, the multiple linear regression was used. The R package “pROC” was used to plot receiver operating characteristic (ROC) curves and calculate the area under the curve (AUC) value and 95% confidence interval (CI). Two-way ANOVA analysis was performed by the R function “aov”. The metabolites with *p* < 0.05 in dosage, pCR, and interaction dimensions were selected independently. The nonparametric Wilcoxon rank-sum test was used to compare the differences in metabolite levels between pCR and non-pCR patients. To calculate the correlation between amino acids, Pearson correlation was performed using the R package “Hmisc”.

### Reporting summary

Further information on research design is available in the Nature Portfolio Reporting Summary linked to this article.

## Supplementary information

Supplementary Information

Description of Additional Supplementary Files

Supplementary Data 1-7

Reporting Summary

## Data Availability

The raw LC−MS data files generated in this study have been deposited in the National Omics Data Encyclopedia under accession code OEP003417 that are publicly available to access . Pathway enrichment analysis was based on KEGG pathway database (https://www.kegg.jp). The sequence data of microbiome data accessed can be accessed at https://ngdc.cncb.ac.cn/gsa/browse/CRA002850. Metabolite annotation and quantification results of the whole dataset were provided in the Supplementary Data 1. The 219 significant metabolites associated with the nCRT treatment were provided in the Supplementary Data 2. Levels of acyl carnitines used for data analysis were provided in Supplementary Data 3. Metabolites associated with hematologic toxicities were provided in Supplementary Data 4. Results of the two-way ANOVA analyses were provided in Supplementary Data 5. Levels of amino acids between pCR and non-pCR patients used for data analysis were in Supplementary Data 6. The details of enrich pathways are listed in Supplementary Data 7. Source data are provided with this paper.

## Source data

Source Data
