# Publisher Correction: Serum metabolic traits reveal therapeutic toxicities and responses of neoadjuvant chemoradiotherapy in patients with rectal cancer

**DOI:** 10.1038/s41467-023-36011-3

**Published:** 2023-01-19

**Authors:** Hongmiao Wang, Huixun Jia, Yang Gao, Haosong Zhang, Jin Fan, Lijie Zhang, Fandong Ren, Yandong Yin, Yuping Cai, Ji Zhu, Zheng-Jiang Zhu

**Affiliations:** 1 Interdisciplinary Research Center on Biology and Chemistry, Shanghai Institute of Organic Chemistry, Chinese Academy of Sciences, Shanghai, 200032 China; 2 University of Chinese Academy of Sciences, Beijing, 100049 China; 3 Department of Ophthalmology, Shanghai General Hospital, Shanghai Jiao Tong University School of Medicine, Shanghai, 200080 China; 4 Department of Biostatistics, Fudan University Shanghai Cancer Center, Shanghai, 200032 China; 5 Cancer Hospital of the University of Chinese Academy of Sciences (Zhejiang Cancer Hospital), Hangzhou, 310005 China; 6 Institute of Cancer and Basic Medicine, Chinese Academy of Sciences, Hangzhou, 310000 China; 7 Zhejiang Key Laboratory of Radiation Oncology, Hangzhou, 310000 China; 8 Department of Radiation Oncology, Fudan University Shanghai Cancer Center, Shanghai, 200032 China; 9 Department of Oncology, Shanghai Medical College, Fudan University, Shanghai, 200031 China; 10 Shanghai Key Laboratory of Radiation Oncology, Shanghai, 200032 China; 11 Shanghai Key Laboratory of Aging Studies, Shanghai, 201210 China

**Keywords:** Metabolomics, Colorectal cancer, Mass spectrometry

Correction to: *Nature Communications* 10.1038/s41467-022-35511-y, published online 17 December 2022

The original version of this manuscript contained a link to the Peer Review file which the authors had opted-out of for publication. This Peer Review file has now been redacted. In addition, the ‘Peer Review Information’ statement has been updated to remove the text “Peer reviewer reports are available.”

