# Supplementary material for: Serum metabolic traits reveal therapeutic toxicities and responses of neoadjuvant chemoradiotherapy in patients with rectal cancer
Source: Nat Commun. 2022 Dec 17;13:7802. doi: 10.1038/s41467-022-35511-y (PMC9759530; doi:10.1038/s41467-022-35511-y)
Supplement: Supplementary file 1 — Supplementary Information [file 41467_2022_35511_MOESM1_ESM.pdf]

Supplementary information for

Serum Metabolic Traits Reveal Therapeutic Toxicities and Responses of Neoadjuvant Chemoradiotherapy in Patients with Rectal Cancer

Supplementary Tables 1-8 in Page S2-S7

Supplementary Figures 1-12 in Page S8-S19

Supplementary Table 1. Demographics of LARC patients from samples used in this study.

Characteristics	Total			pCR			non-pCR		
**Sex**	**Male (n=117)**	**Female (n=48)**	**All (n=165)**	**Male (n=26)**	**Female (n=12)**	**All (n=38)**	**Male (n=84)**	**Female (n=32)**	**All (n=116)**
Age (mean±s.d., year)	53.48±10.76	51.96±10.01	53.04±10.57	54.23±14.32	46.08±12.04	51.66±14.16	53.12±9.40	54.06±8.57	53.38±9.19
BMI (mean±s.d.)	23.11±2.80	22.82±3.30	23.03±2.96	23.74±3.45	22.02±2.51	23.18±3.28	22.93±2.51	22.87±3.47	22.91±2.81
Stage (II/III/IV)									
II	3	1	4	0	0	0	3	1	4
III	93	36	129	20	10	30	67	24	91
IV	16	8	24	5	2	7	10	4	14
Distance from anal verge (mean±s.d., cm)	5.07±1.78	5.52±1.86	5.21±1.81	4.88±1.63	5.88±1.69	5.21±1.72	5.20±1.83	5.39±1.93	5.25±1.85
UGT1A1 genotype									
*1*1	69	32	101	14	7	21	50	22	72
*1*28	25	6	31	9	3	12	16	2	18
No. of irinotecan cycles (weekly courses, mean±s.d.)	3.69±1.14	3.31±0.96	3.58±1.10	3.69±1.10	3.42±1.04	3.58±1.10	3.75±1.14	3.19±0.92	3.59±1.11
Diarrhea									
0	25	5	30	8	2	10	16	3	19
1	5	1	6	3	0	3	2	1	3
2	4	4	8	0	1	1	4	3	7
3	4	7	11	1	4	5	3	2	5
Hematologic toxicity index (lowest cell counts or level, mean±s.d.)									
White blood cells (x10^9/L)	2.85±1.11	2.34±0.94	2.69±1.09	2.74±1.10	2.66±0.93	2.72±1.06	2.97±1.06	2.13±0.87	2.71±1.07
Neutrophils (x10^9/L)	1.87±0.74	1.46±0.64	1.75±0.74	1.79±0.73	1.61±0.55	1.74±0.69	1.95±0.71	1.33±0.58	1.76±0.73
Blood platelet cells (x10^9/L)	142.91±41.40	144.95±48.74	143.53±43.79	135.32±32.57	140.10±37.28	136.69±34.05	145.54±44.54	144.64±52.19	145.27±46.98
Hemoglobin (g/L)	116.34±15.93	98.71±14.63	110.93±17.54	117.36±15.11	106.10±13.00	114.14±15.40	115.88±15.58	95.82±14.90	109.84±17.92

Supplementary Table 2. The selected predictors for diarrhea prediction.

Predictor	Coefficient[1]	p value[2]
Serine	-1.30	0.0064
Phenylalanine	0.98	0.0142
Uridine	-1.07	0.0292
Sex	1.17	0.0053

Note: 1, refers to the coefficient of each predictor in the logistic model. 2, refers to the p value of each predictor in the model (z-test of logistic regression).

Prediction equation:

$$Logit(P) = -0.14 - 1.07 \times M_{Uridine} - 1.30 \times M_{Serine} + 0.98 \times M_{Phenylalaine} + 1.17 \times Sex$$

Supplementary Table 3. Power of the factors for diarrhea prediction

Predictor	Number of group A	Number of group B	Power
Serine	30	25	0.97
Phenylalanine	30	25	0.86
Urdine	30	25	0.91
Sex	30	25	0.94

Supplementary Table 4. Metabolites used for predicting the lowest cell count of white blood cells.

Label	Metabolite name	KEGG ID	Coefficient[1]	p value[2]
M_1	1,1-Dimethyl-4-phenylpiperazinium	NA	-0.88	0.0110
M_2	Thyroxine	C01829	-0.67	0.0273
M_3	Ubiquinone (Q2)	NA	-0.60	0.0416
M_4	PE (38:6)	NA	-0.41	0.0143
M_5	Vaccenic acid	NA	-0.29	0.0376
M_6	Gulonic gamma-lactone	C01040	-0.28	0.0092
M_7	Malonic acid	C00383	-0.15	0.0033
M_8	4-Vinylphenol	C05627	-0.10	0.0091
M_9	Nicotine	C00745	0.14	0.0034
M_{10}	PC (36:3)	NA	0.16	0.0368
M_{11}	Car (11:0)	NA	0.20	0.0056
M_{12}	PC (O-30:2)	NA	0.21	0.0420
M_{13}	Tartaric acid	C00552	0.23	0.0409
M_{14}	Glyceraldehyde	C02154	0.23	0.0236
M_{15}	LPE (18:3)	NA	0.29	0.0106

Note: 1, refers to the coefficient of one metabolite in the multiple linear regression model. 2, refers to the two-sided Student's t-test p value of Pearson correlation for each metabolite.

Prediction equation:

$$WBC_{predict} = 46.04 - 0.88 \times M_1 - 0.67 \times M_2 - 0.60 \times M_3 - 0.41 \times M_4 - 0.29 \times M_5 - 0.28 \times M_6 - 0.15 \times M_7 - 0.10 \times M_8 + 0.14 \times M_9 + 0.16 \times M_{10} + 0.20 \times M_{11} + 0.21 \times M_{12} + 0.23 \times M_{13} + 0.23 \times M_{14} + 0.29 \times M_{15}$$

Supplementary Table 5. Metabolites used for predicting the lowest cell count of neutrophils.

Label	Metabolite name	KEGG ID	Coefficient[1]	p value[2]
M_1	1,1-Dimethyl-4-phenylpiperazinium	NA	-0.66	0.0024
M_2	Malonic acid	C00383	-0.26	0.0001
M_3	Thyroxine	C01829	-0.25	0.0033
M_4	Threonine	C00188	-0.22	0.0318
M_5	SM (d32:1)	NA	-0.22	0.0008
M_6	Vaccenic acid	NA	-0.18	0.0443
M_7	Gulonic gamma-lactone	C01040	-0.16	0.0337
M_8	3-(Pyrazol-1-yl)-alanine	C01162	0.05	0.0229
M_9	Nicotine	C00745	0.07	0.0075
M_{10}	Car (11:0)	NA	0.13	0.0115
M_{11}	Tartaric acid	C00552	0.18	0.0364
M_{12}	LPE (18:3)	NA	0.21	0.0158

Note: 1, refers to the coefficient of one metabolite in the multiple linear regression model. 2, refers to the two-sided Student's t-test p value of Pearson correlation for each metabolite.

Prediction equation:

$$NEUT_{predict} = 32.45 - 0.66 \times M_1 - 0.26 \times M_2 - 0.25 \times M_3 - 0.22 \times M_4 - 0.22 \times M_5 - 0.18 \times M_6 - 0.16 \times M_7 + 0.05 \times M_8 + 0.07 \times M_9 + 0.13 \times M_{10} + 0.18 \times M_{11} + 0.21 \times M_{12}$$

Supplementary Table 6. Metabolites used for predicting the lowest level of hemoglobin.

Label	Metabolite name	KEGG ID	Coefficient[1]	p value[2]
M_1	Cellobiose	C00185	-6.84	0.0171
M_2	PC (P-38:4)	NA	-4.11	0.0133
M_3	Alpha-N-Phenylacetyl-glutamine	C04148	-1.78	0.0334
M_4	Salicylic acid	C00805	1.81	0.0353
M_5	Acetaminophen glucuronide	NA	2.60	0.0327
M_6	Car (9:1)	NA	3.80	0.0069
M_7	LPA (18:2)	NA	4.22	0.0069
M_8	Uridine	C00299	5.11	0.0284
M_9	Hydroxyphenyllactic acid	C03672	6.45	0.0005
M_{10}	S1P (d18:2)	NA	7.41	0.0083
M_{11}	Asparagine	C00152	8.29	0.0009
M_{12}	2-Hydroxyquinoline	C06338	12.11	0.0113

Note: 1, refers to the coefficient of one metabolite in the multiple linear regression model. 2, refers to the two-sided Student's t-test p value of Pearson correlation for each metabolite.

Prediction equation:

$$\mathrm{Hb_{predict}} = -760.50 - 6.84 \times M_1 - 4.11 \times M_2 - 1.78 \times M_3 + 1.81 \times M_4 + 2.60 \times M_5 + 3.80 \times M_6 + 4.22 \times M_7 + 5.11 \times M_8 + 6.45 \times M_9 + 7.41 \times M_{10} + 8.29 \times M_{11} + 12.11 \times M_{12} - 12.46 \times Sex$$

Supplementary Table 7. Metabolites used for predicting the lowest cell count of blood platelet cells.

Label	Metabolite name	KEGG ID	Coefficient[1]	p value[2]
M_1	N-Acetylalanine	NA	-28.12	0.0054
M_2	Betaine	C00719	-26.46	0.0403
M_3	3-Hydroxyvaleric acid	NA	-17.64	0.0204
M_4	PC (40:6)	NA	-17.49	0.0060
M_5	Targinine	C03884	-16.79	0.0045
M_6	Nervonic acid	C08323	-15.92	0.0019
M_7	Bilirubin	C00486	-13.78	0.0038
M_8	Kynurenine	C00328	-13.73	0.0017
M_9	N-Acetylneuraminic acid	C00270	-11.84	0.0152
M_{10}	LPE (20:5)	NA	-11.60	0.0398
M_{11}	Allose	C01487	-8.97	0.0024
M_{12}	LPC (17:1)	NA	-8.42	0.0490
M_{13}	PC (36:5)	NA	-7.95	0.0363
M_{14}	1,3,7-Trimethyluric acid	C16361	-7.11	0.0250
M_{15}	Dehydroisoandrosterone sulfate	C04555	7.10	0.0201
M_{16}	PC (34:2)	NA	20.91	0.0149
M_{17}	Pseudouridine	C02067	40.78	0.0405

Note: 1, refers to the coefficient of one metabolite in the multiple linear regression model. 2, refers to the two-sided Student's t-test p value of Pearson correlation for each metabolite.

Prediction equation:

$$BPC_{predict} = 3170.64 - 28.12 \times M_1 - 26.46 \times M_2 - 17.64 \times M_3 - 17.49 \times M_4 - 16.79 \times M_5 - 15.92 \times M_6 - 13.78 \times M_7 - 13.73 \times M_8 - 11.84 \times M_9 - 11.60 \times M_{10} - 8.97 \times M_{11} - 8.42 \times M_{12} - 7.95 \times M_{13} - 7.11 \times M_{14} + 7.10 \times M_{15} + 20.91 \times M_{16} + 40.78 \times M_{17}$$

Supplementary Table 8 Summary of omics studies on chemo- and radio- therapies

Cancer type	Key findings	Omics technology	Clinical therapy	Reference
Rectal cancer	Identified 15 potential metabolite biomarkers to predict tumor response to neoadjuvant chemo-radiation therapy at baseline in patients with locally advanced rectal cancer.	LC-MS based untargeted metabolomics	Neoadjuvant chemoradiotherapy	Jia *et al.*, 2018, *Radiother Oncol.*[1]
Rectal cancer	Identified ten microbiota biomarkers such as *Dorea*, *Anaerostipes*, and *Streptococcus* for the response-prediction classifier.	16S rRNA sequencing	Neoadjuvant chemoradiotherapy	Yi *et al.*, 2021, *Clin. Cancer Res.*[2]
Rectal cancer	Identified that patients with pCR had lower level of valine at baseline and those with relapse had lower level of succinate.	GC-MS based targeted metabolomics	Neoadjuvant chemoradiotherapy	Rodriguez *et al.*, 2021, *PLoS One*[3]
Breast cancer	Showed changes in serum metabolites during chemotherapy treatment and revealed metabolic differences between survivors and nonsurvivors in tissue samples.	NMR-based untargeted metabolomics	Neoadjuvant chemotherapy	Debik *et al.*, 2019, *J. Proteome. Res.*[4]
Breast cancer	Identified two proteins of proline biosynthesis pathway, PYCR1 and ALDH18A1, that were significantly associated with resistance to treatment.	Proteomics	Neoadjuvant chemotherapy	Shenoy *et al.*, 2020, *Mol. Syst. Biol.*[5])
Breast cancer	Identified higher levels of decosahexaenoic acid and secondary bile acids in responders. Found that glycohyocholic acid and glycodeoxycholic acid can classify triple-negative patients regarding treatment response.	LC-MS based untargeted metabolomics	Neoadjuvant chemotherapy	Diaz *et al.*, 2022, *Mol. Oncol.*[6]
Esophageal squamous cell carcinoma	Identified numerous differentially expressed genes and miRNAs from nCRT responder group.	Transcriptomics	Neoadjuvant chemoradiotherapy	Wang *et al.*, 2021 *Front Pharmacol.*[7]

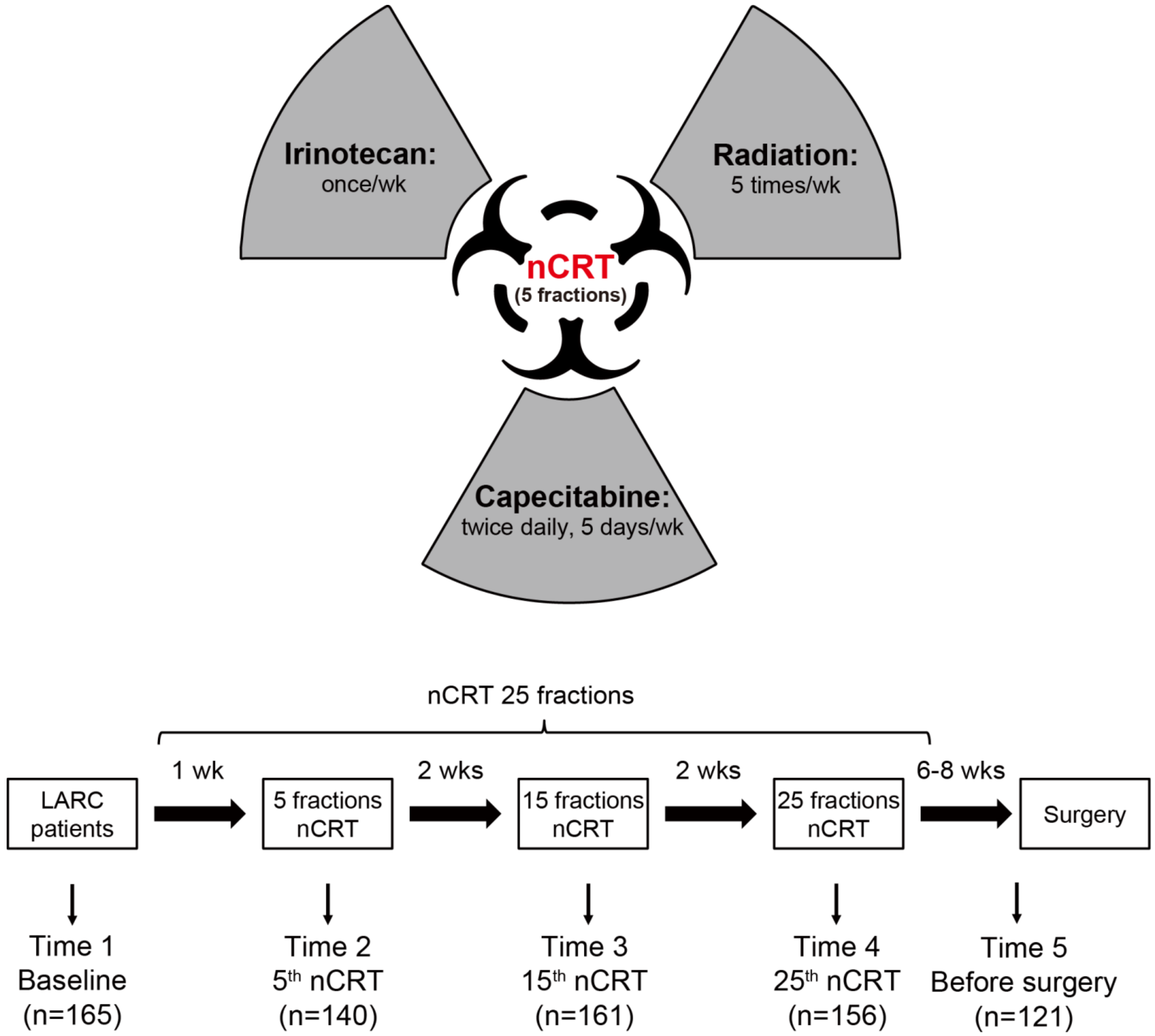

Supplementary Figure 1. Scheme of the nCRT treatment. The number below each time point represents the number of serum samples collected for metabolomics analysis.

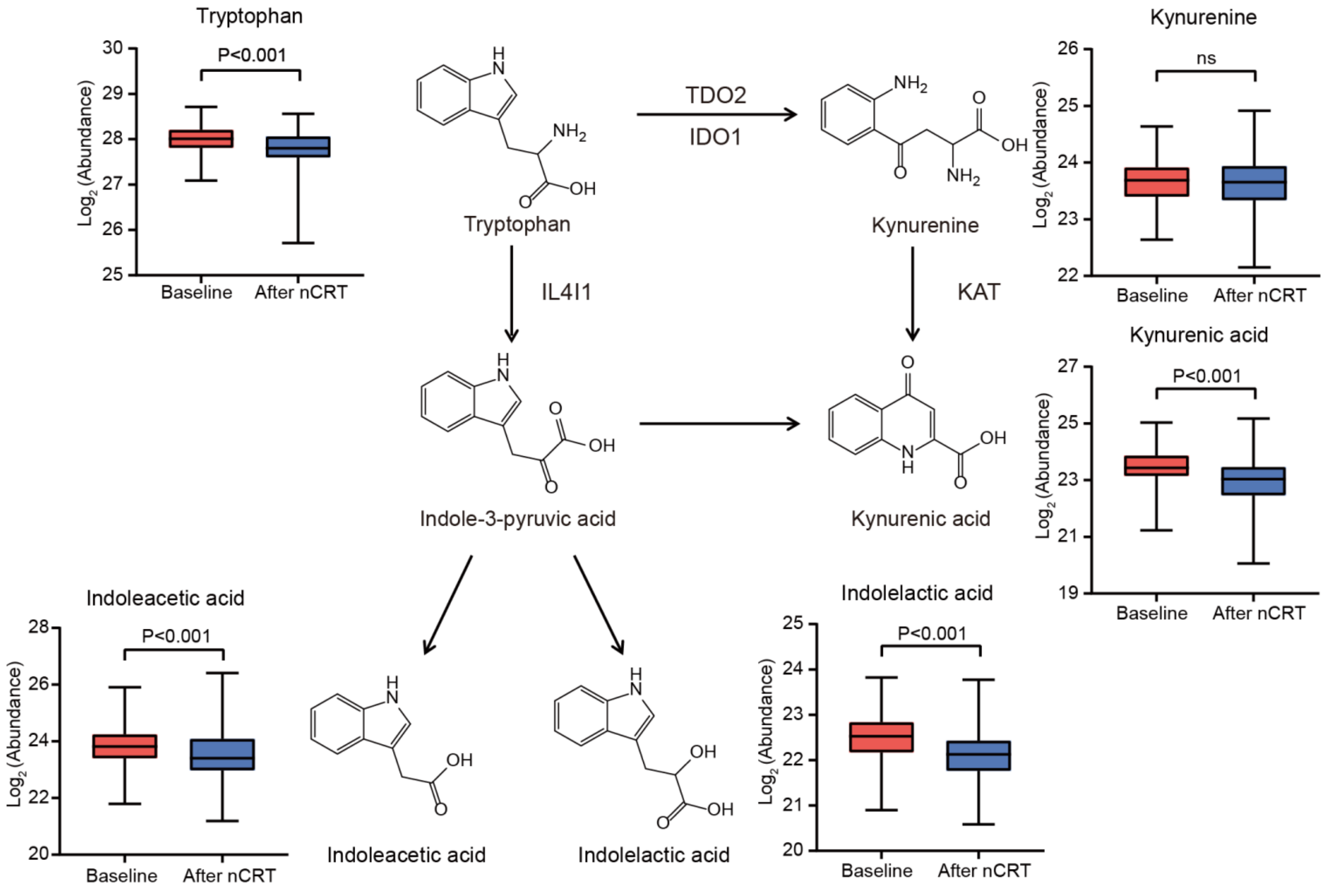

Supplementary Figure 2. Levels of metabolites in tryptophan metabolism before (baseline, n=165) and after nCRT (25th nCRT, n=156) (Two-sided Wilcoxon test; ns, not significant). The centerline of boxes depicts the median values; the bottom and top box edges correspond to the first and third quartiles, and whiskers indicated the minimum and maximum values.

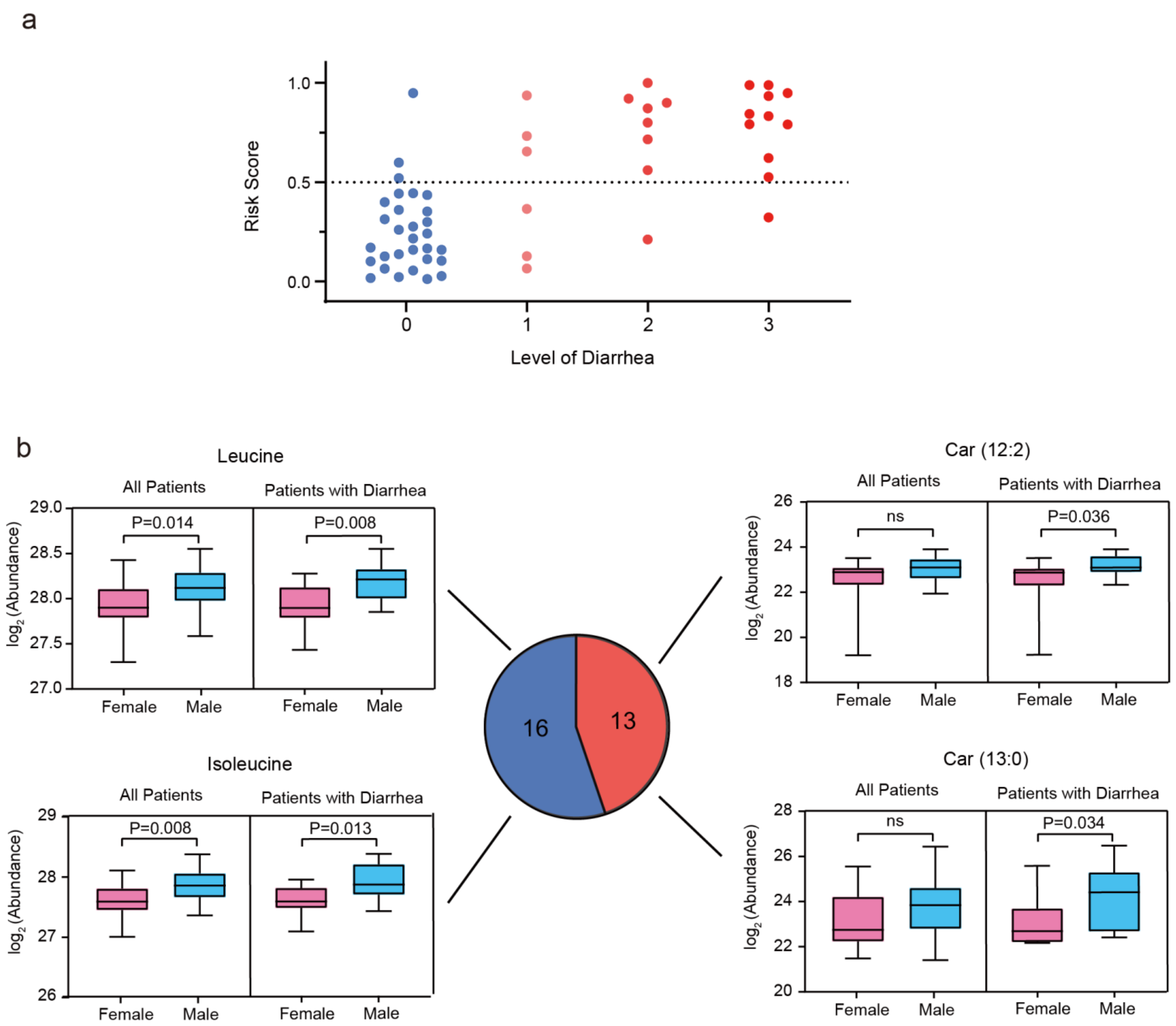

Supplementary Figure 3. Prediction model of diarrhea and the diarrhea related metabolites. (**a**) Performance of the diarrhea prediction model for predicting different levels (Level 0 (No diarrhea): n=30; Level 1: n=6; Level 2: n=8; Level 3: n=11) of diarrhea in LARC patients. (**b**) Pie chart: the 29 metabolites showed significant difference between male (n=13) and female (n=12) in patients with diarrhea. 16 metabolites with significant differences between females (n=17) and males (n=38) both in all patients and in patients with diarrhea (blue in pie chart). Left boxplots showed levels of leucine and isoleucine between females and males in all patients and patients with diarrhea. 13 metabolites with significant differences only between female and male patients with diarrhea (red in pie chart). Right boxplots showed levels of Car (12:2) and Car (13:0) between females and males in all patients and patients with diarrhea (Two-sided Wilcoxon test; ns, not significant). The centerline of boxes depicts the median values; the bottom and top box edges correspond to the first and third quartiles, and whiskers indicated the minimum and maximum values.

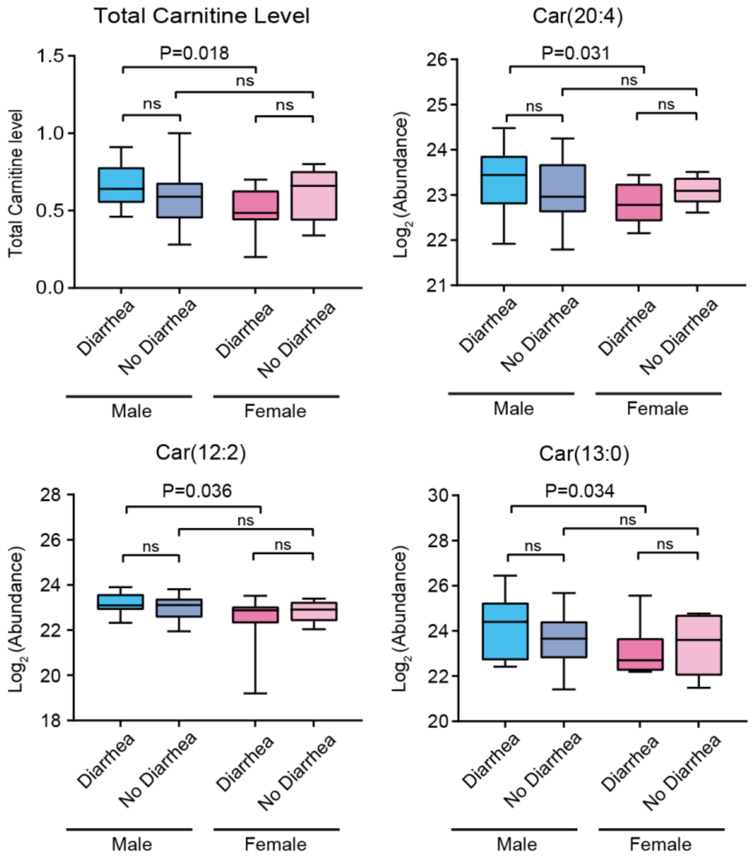

Supplementary Figure 4. Levels of total carnitine, Car (20:4), Car (12:2), and Car (13:0) between males with diarrhea (n=13), males without diarrhea (n=25), females with diarrhea (n=12), females without diarrhea (n=5) (Two-sided Wilcoxon test; ns, not significant). The centerline of boxes depicts the median values; the bottom and top box edges correspond to the first and third quartiles, and whiskers indicated the minimum and maximum values.

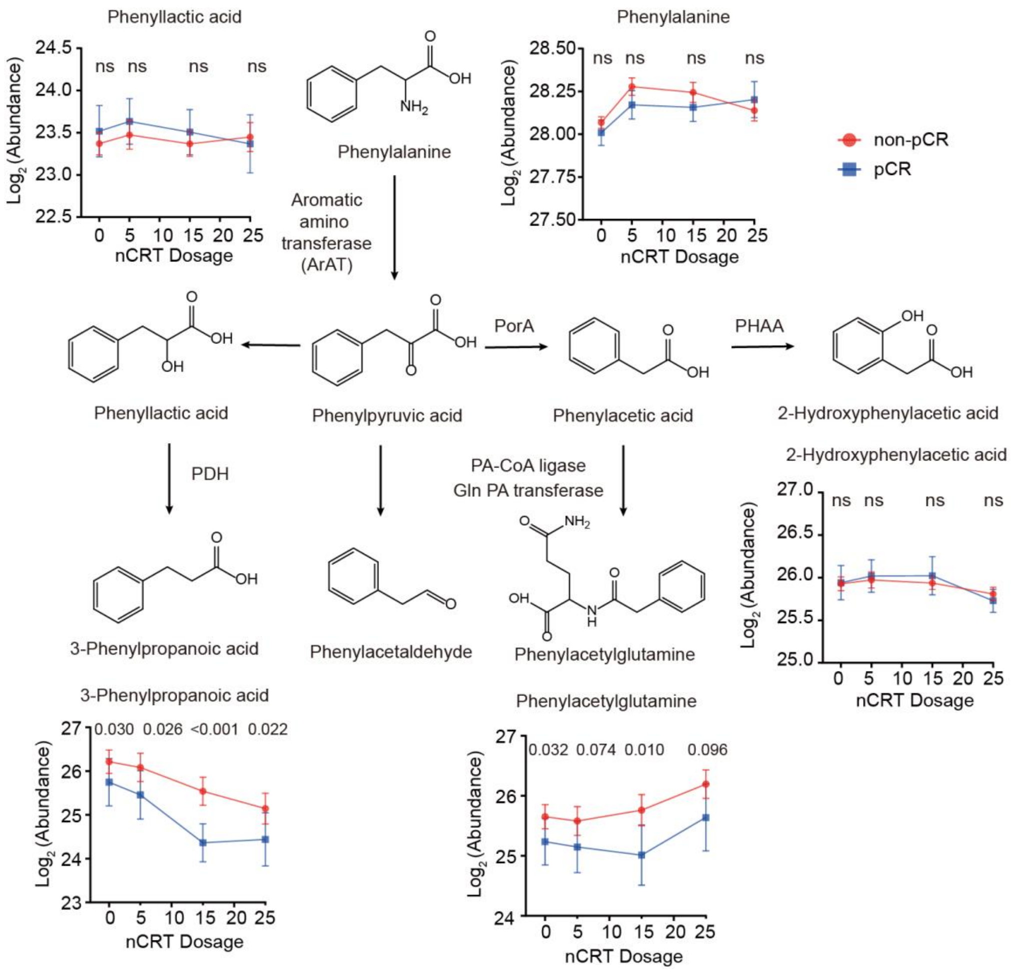

Supplementary Figure 5. Levels of metabolites in phenylalanine metabolism for pCR and non-pCR patients (At individual time points, n=38, 35, 37, 38 for pCR and n=116, 96, 113, 116 for non-pCR) over the course of nCRT treatment (Two-sided Wilcoxon test; ns, not significant). The dot depicts the mean values. Error bars represent 95% confidence intervals.

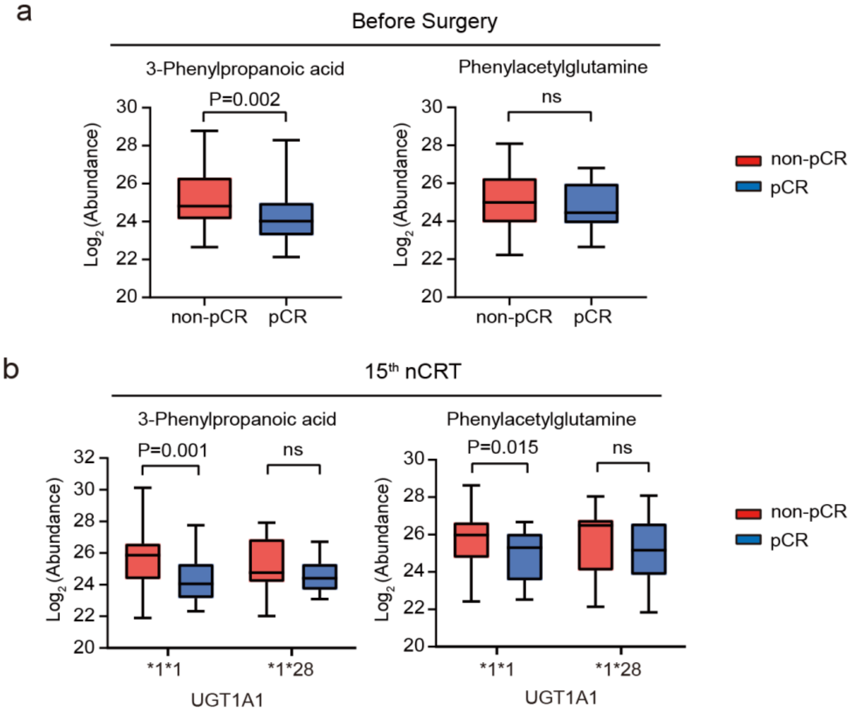

Supplementary Figure 6. Levels of 3-phenylpropanoic acid and phenylacetylglutamine for pCR and non-pCR patients. (**a**) Levels of 3-phenylpropanoic acid and phenylacetylglutamine at Time 5 (after rest and before surgery) between pCR (n=30) and non-pCR (n=84) patients (Two-sided Wilcoxon test; ns, not significant). (**b**) Levels of 3-phenylpropanoic acid and phenylacetylglutamine between pCR and non-pCR patients in different *UGT1A1* genotypes (*1*1: pCR, n=21, non-pCR, n=72; *1*28: pCR, n=11, non-pCR, n=16) after the 15th nCRT (Two-sided Wilcoxon test; ns, not significant). The centerline of boxes depicts the median values; the bottom and top box edges correspond to the first and third quartiles, and whiskers indicated the minimum and maximum values.

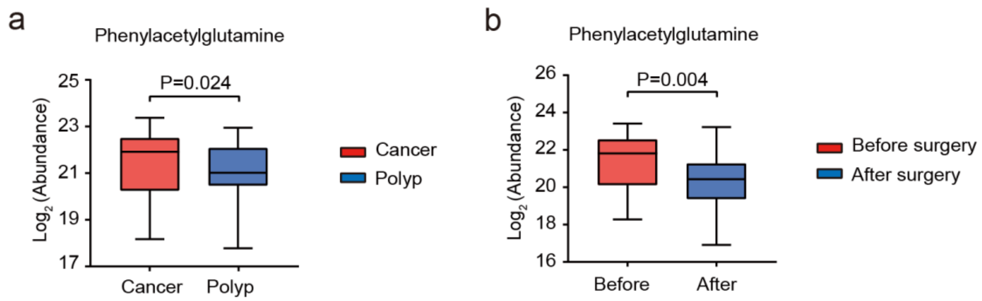

Supplementary Figure 7. Levels of pheylacetylglutamine (PAGln) in plasma samples of CRC patients. (a) Boxplots of phenylacetylglutamine abundances in plasma samples between patients with colorectal cancer (cancer; n=73) and benign polyp (polyp; n=73) (Two-sided Wilcoxon Test); (b) Boxplots of phenylacetylglutamine abundances in plasma samples for colorectal cancer patients before and after surgery (n=33; Two-sided paired Wilcoxon Test). The centerline of boxes depicts the median values; the bottom and top box edges correspond to the first and third quartiles, and whiskers indicated the minimum and maximum values. The data was retrieved from a previously published work[8].

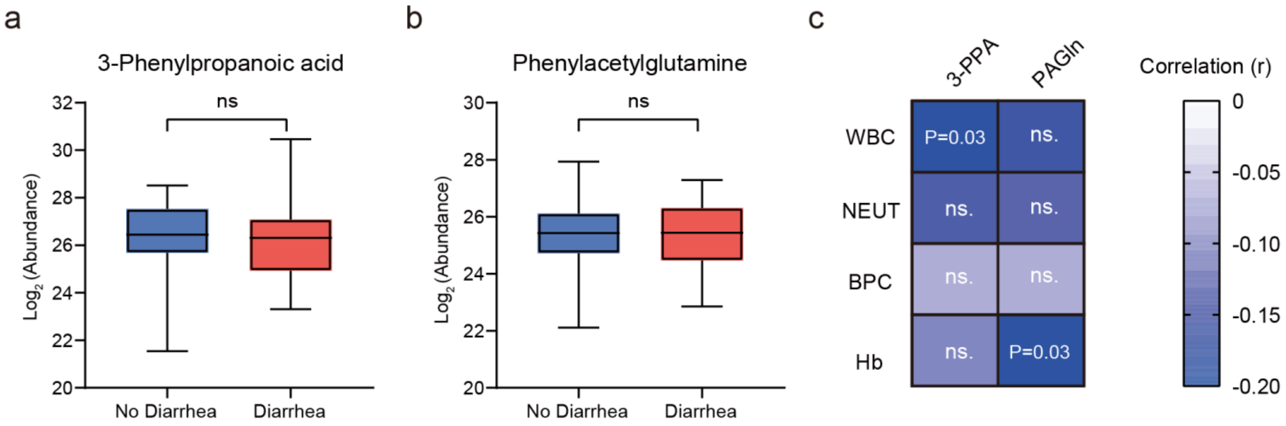

Supplementary Figure 8. Boxplots of (**a**) 3-phenylpropanois acid (3-PPA) and (**b**) phenylacetylglutamine (PAGln) abundances between patients with diarrhea and without diarrhea at baseline (Two-sided Wilcoxon test; ns, not significant). The centerline of boxes depicts the median values; the bottom and top box edges correspond to the first and third quartiles, and whiskers indicated the minimum and maximum values. (**c**) Correlations between pre-therapeutic serum levels of 3-PPA and PAGln and cell counts that indicate hematologic toxicity (Pearson correlation; ns, not significant). The p-value of Pearson correlation coefficient was calculated by two-sided Student's t-test. WBC, white blood cells; NEUT, neutrophils; BPC, blood platelet cells; Hb, hemoglobin.

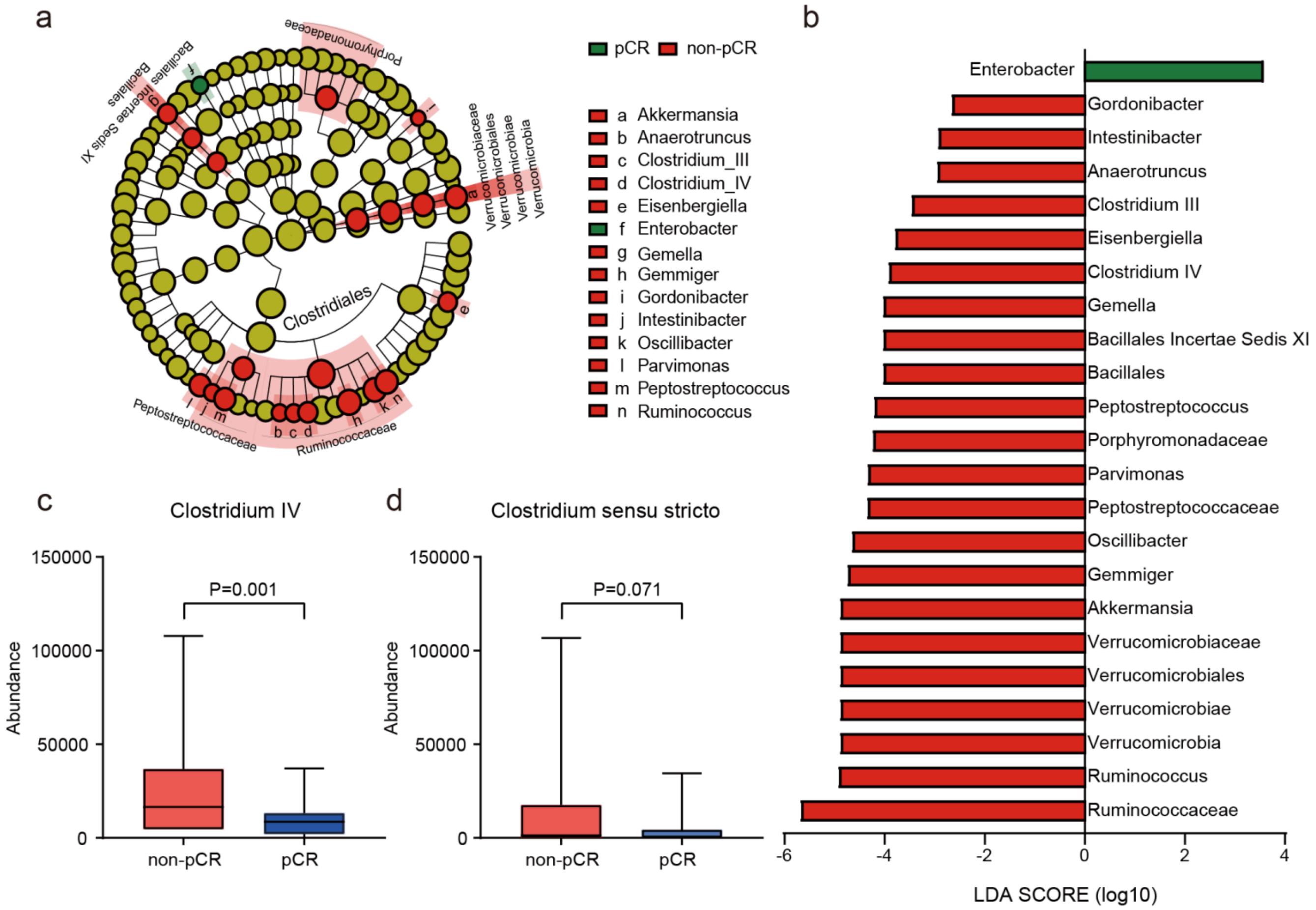

Supplementary Figure 9. Cladogram (**a**) and histogram (**b**) of significantly different taxa between pCR (n=26) and non-pCR (n=46) patients at baseline using the Linear Discriminant Analysis (LDA) (Kruskal-Wallis test, $P < 0.05$, LDA score > 2). Green and red labels represent taxa abundances that were higher in pCR and non-pCR patients. Boxplots of (**c**) *Clostridium IV* and (**d**) *Clostridium sensu stricto* abundances between pCR (n=26) and non-pCR (n=46) patients at baseline (Two-sided Welch's test). The centerline of boxes depicts the median values; the bottom and top box edges correspond to the first and third quartiles, and whiskers indicated the minimum and maximum values.

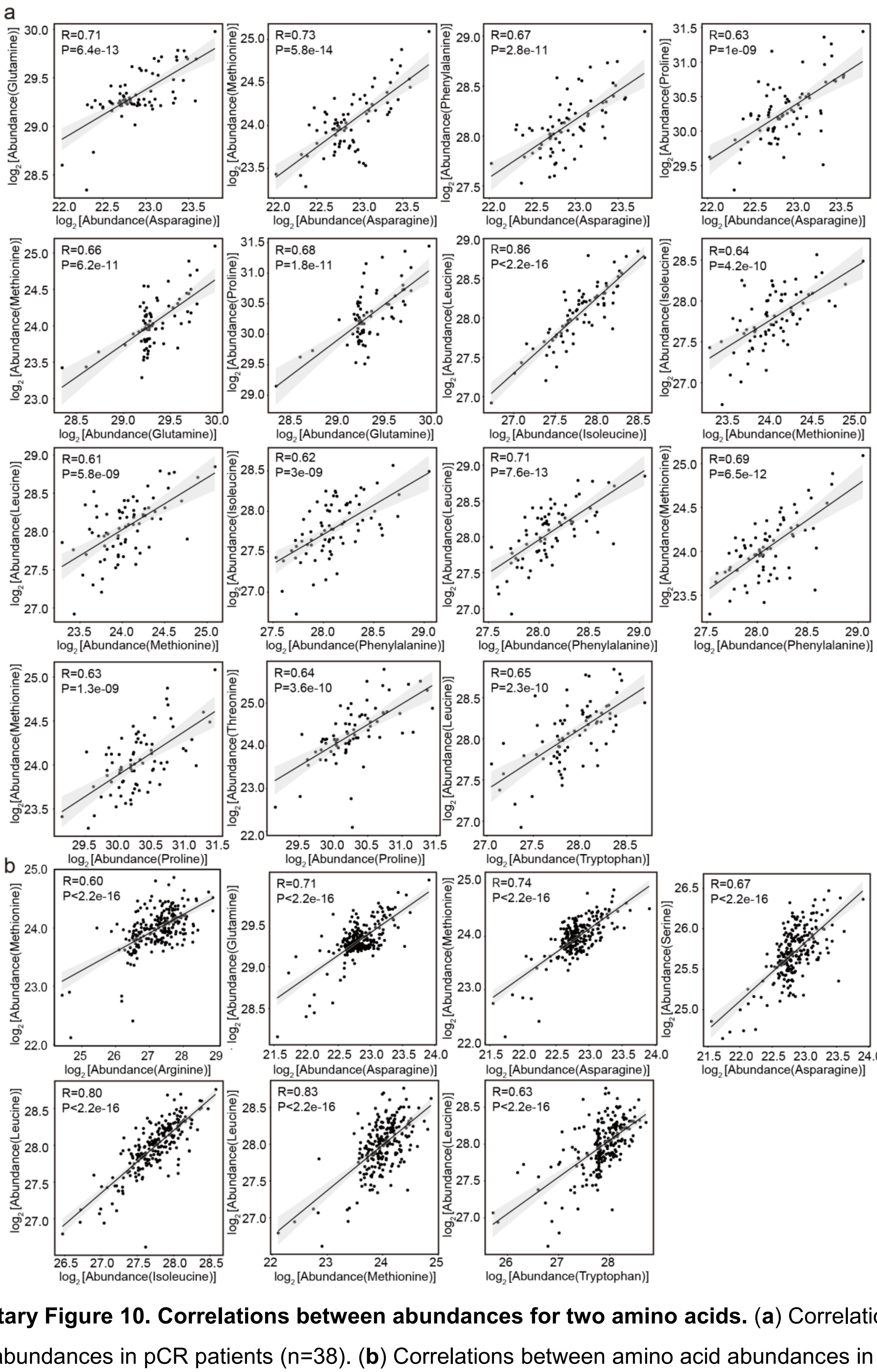

Supplementary Figure 10. Correlations between abundances for two amino acids. (**a**) Correlations between amino acid abundances in pCR patients (n=38). (**b**) Correlations between amino acid abundances in non-pCR patients (n=116). The p-value of Pearson correlation coefficient was calculated by two-sided Student's t-test. Error bands represent 95% confidence intervals.

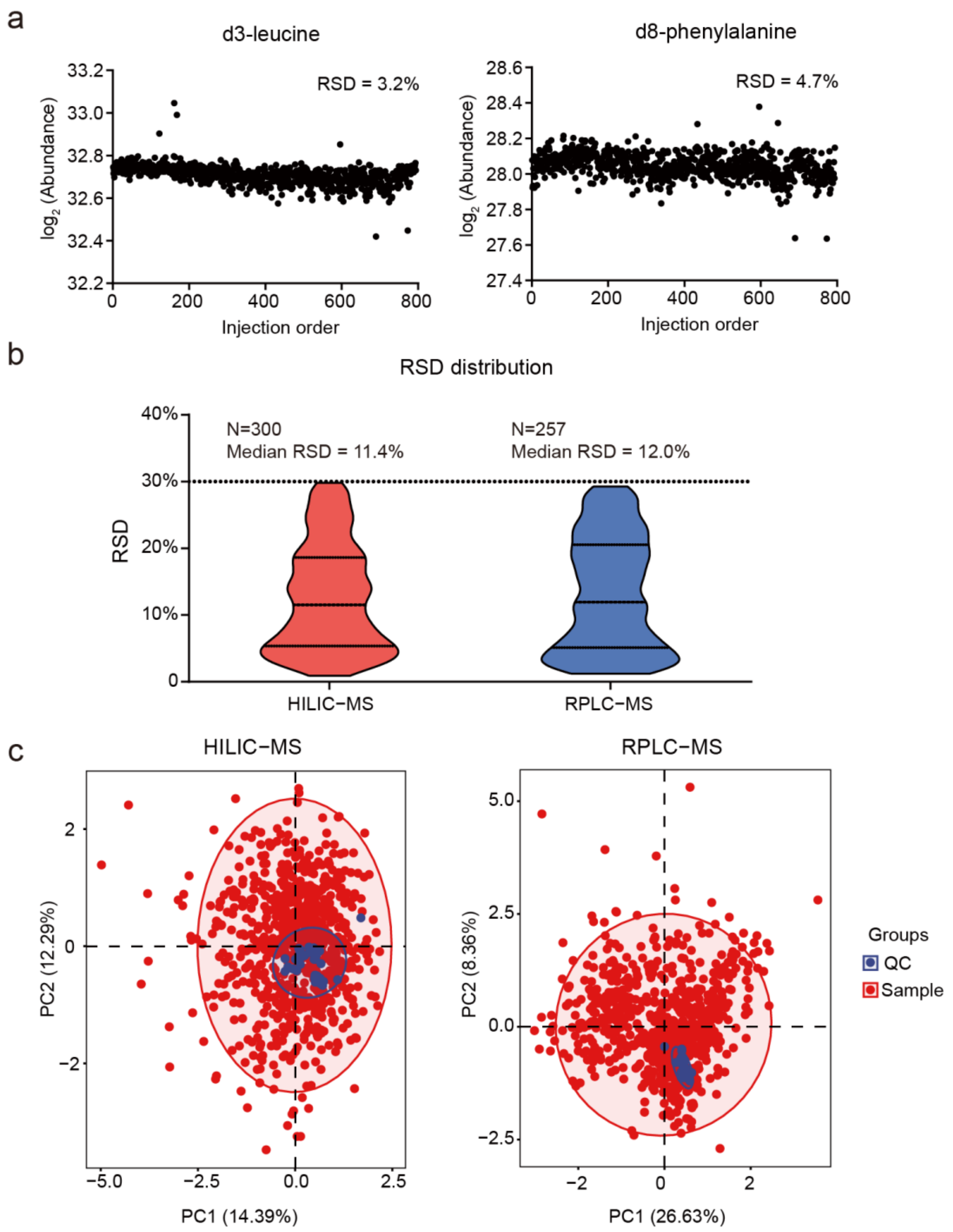

Supplementary Figure 11. Quality control of metabolomics data acquisition. (**a**) Peak areas of d3-leucine (left) and d8-phenylalanine (right) over the sample injection order during data acquisition. (**b**) Distribution of relative standard deviations (RSDs) of metabolites for HILIC−MS and RPLC−MS measurements. (**c**) PCA score plots for metabolomics data in HILIC−-MS and RPLC−MS measurements.

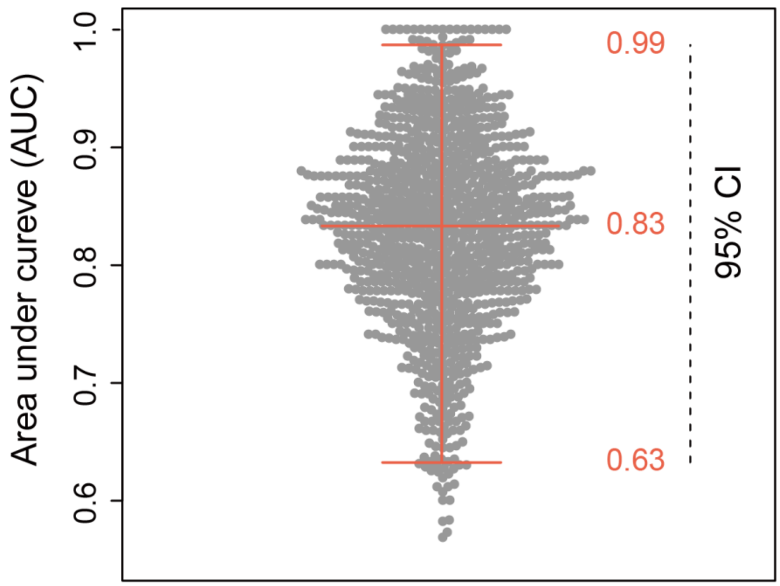

Supplementary Figure 12. Distribution of AUC values for the 1000 times of bootstrap sampling for the diarrhea prediction model (Diarrhea: n=25; No diarrhea: n=30). The centerlines of the box plots indicate the mean values and the lower and upper lines correspond to 2.5th and 97.5th quartiles, indicating the 95% range of AUC values.

Supplementary References

1. Jia H*, et al.* Predicting the pathological response to neoadjuvant chemoradiation using untargeted metabolomics in locally advanced rectal cancer. *Radiotherapy and oncology* **128**, 548-556 (2018).
2. Yi Y*, et al.* Gut Microbiome Components Predict Response to Neoadjuvant Chemoradiotherapy in Patients with Locally Advanced Rectal Cancer: A Prospective, Longitudinal Study. *Clinical cancer research* **27**, 1329-1340 (2021).
3. Rodriguez-Tomas E*, et al.* Identification of potential metabolic biomarkers of rectal cancer and of the effect of neoadjuvant radiochemotherapy. *PloS one* **16**, e0250453 (2021).
4. Debik J*, et al.* Assessing Treatment Response and Prognosis by Serum and Tissue Metabolomics in Breast Cancer Patients. *Journal of proteome research* **18**, 3649-3660 (2019).
5. Shenoy A*, et al.* Proteomic patterns associated with response to breast cancer neoadjuvant treatment. *Molecular systems biology* **16**, e9443 (2020).
6. Diaz C*, et al.* Predicting dynamic response to neoadjuvant chemotherapy in breast cancer: a novel metabolomics approach. *Molecular oncology* **16**, 2658-2671 (2022).
7. McQuade RM, Stojanovska V, Abalo R, Bornstein JC, Nurgali K. Chemotherapy-Induced Constipation

and Diarrhea: Pathophysiology, Current and Emerging Treatments. *Front Pharmacol* **7**, 414 (2016).

8. Wang Z*, et al.* Development of a Correlative Strategy To Discover Colorectal Tumor Tissue Derived Metabolite Biomarkers in Plasma Using Untargeted Metabolomics. *Analytical chemistry* **91**, 2401-2408 (2019).
