# Supplementary material for: Serum metabolic traits reveal therapeutic toxicities and responses of neoadjuvant chemoradiotherapy in patients with rectal cancer
Source: Nat Commun. 2022 Dec 17;13:7802. doi: 10.1038/s41467-022-35511-y (PMC9759530; doi:10.1038/s41467-022-35511-y)
Supplement: Supplementary file 2 — Description of Additional Supplementary Files [file 41467_2022_35511_MOESM2_ESM.docx]

File Name: Supplementary Data 1

Description: Metabolite annotation and quantification results of the whole dataset.

File Name: Supplementary Data 2

Description: The 219 significantly changed metabolites associated with the nCRT treatment.

File Name: Supplementary Data 3

Description: Levels of acyl carnitines used for data analysis.

File Name: Supplementary Data 4

Description: Metabolites associated with hematologic toxicities.

File Name: Supplementary Data 5

Description: Results of the two-way ANOVA analyses.

File Name: Supplementary Data 6

Description: Levels of amino acids between pCR and non-pCR patients used for data analysis.

File Name: Supplementary Data 7

Description: The details of pathway enrichment results.
