# Supplementary material for: Serum metabolic traits reveal therapeutic toxicities and responses of neoadjuvant chemoradiotherapy in patients with rectal cancer
Source: Nat Commun. 2022 Dec 17;13:7802. doi: 10.1038/s41467-022-35511-y (PMC9759530; doi:10.1038/s41467-022-35511-y)
Supplement: Supplementary file 4 — Reporting Summary [file 41467_2022_35511_MOESM4_ESM.pdf]

nature portfolio

Corresponding author(s): Dr. Zheng-Jiang Zhu

Last updated by author(s): Nov 25, 2022

Reporting Summary

Nature Portfolio wishes to improve the reproducibility of the work that we publish. This form provides structure for consistency and transparency in reporting. For further information on Nature Portfolio policies, see our Editorial Policies and the Editorial Policy Checklist.

Statistics

For all statistical analyses, confirm that the following items are present in the figure legend, table legend, main text, or Methods section.

n/a	Confirmed	
☐	☒	The exact sample size (*n*) for each experimental group/condition, given as a discrete number and unit of measurement
☐	☒	A statement on whether measurements were taken from distinct samples or whether the same sample was measured repeatedly
☐	☒	The statistical test(s) used AND whether they are one- or two-sided<br>*Only common tests should be described solely by name; describe more complex techniques in the Methods section.*
☐	☒	A description of all covariates tested
☐	☒	A description of any assumptions or corrections, such as tests of normality and adjustment for multiple comparisons
☐	☒	A full description of the statistical parameters including central tendency (e.g. means) or other basic estimates (e.g. regression coefficient) AND variation (e.g. standard deviation) or associated estimates of uncertainty (e.g. confidence intervals)
☐	☒	For null hypothesis testing, the test statistic (e.g. *F*, *t*, *r*) with confidence intervals, effect sizes, degrees of freedom and *P* value noted<br>*Give P values as exact values whenever suitable.*
☒	☐	For Bayesian analysis, information on the choice of priors and Markov chain Monte Carlo settings
☒	☐	For hierarchical and complex designs, identification of the appropriate level for tests and full reporting of outcomes
☐	☒	Estimates of effect sizes (e.g. Cohen's *d*, Pearson's *r*), indicating how they were calculated

Our web collection on statistics for biologists contains articles on many of the points above.

Software and code

Policy information about availability of computer code

Data collection	Xcalibur (Version 4.4.16.14, Thermo Fisher Scientific, USA) was used to collect metabolomics data.
Data analysis	Data analyses were performed using publicly available software: MetDNA2 (http://metdna.zhulab.cn/; version 1.1.18), MetFlow (http://metflow.zhulab.cn/ ; version 1.2),G*Power (version 3.1.9.4), MetaboAnalyst (https://www.metaboanalyst.ca; version 5.0), FLASH (version 1.2.8), fqtrim (version 0.9.4), Vsearch (version 2.11.1), and R (version 3.6.1), R package "xcms" (version 3.12.0),"pheatmap" (version 1.0.12),"glmnet" (version 4.0.2), MASS (v7.3.54), phyloseq (version 1.38.0), ggtree (version 3.2.1).

For manuscripts utilizing custom algorithms or software that are central to the research but not yet described in published literature, software must be made available to editors and reviewers. We strongly encourage code deposition in a community repository (e.g. GitHub). See the Nature Portfolio guidelines for submitting code & software for further information.

Data

Policy information about availability of data

All manuscripts must include a data availability statement. This statement should provide the following information, where applicable:

- Accession codes, unique identifiers, or web links for publicly available datasets
- A description of any restrictions on data availability
- For clinical datasets or third party data, please ensure that the statement adheres to our policy

The raw LC-MS data files generated in this study have been deposited in the National Omics Data Encyclopedia under accession code OEP003417 that are accessible at https://www.biosino.org/node. Pathway enrichment analysis was based on KEGG pathway database (https://www.kegg.jp). Metabolite annotation and

March 2021

quantification results of the whole dataset were provided in the Supplementary Data 1. The 219 significant metabolites associated with the nCRT treatment were provided in the Supplementary Data 2. Levels of acyl carnitines used for data analysis were provided in Supplementary Data 3. Metabolites associated with hematologic toxicities were provided in Supplementary Data 4. Results of the two-way ANOVA analyses were provided in Supplementary Data 5. Levels of amino acids between pCR and non-pCR patients used for data analysis were in Supplementary Data 6. The details of enrich pathways are listed in Supplementary Data 7. Source data are provided with this paper.

Human research participants

Policy information about studies involving human research participants and Sex and Gender in Research.

Reporting on sex and gender	We have reported the sex information on the enrolled patients, which is determined by self-reporting (117 males and 48 females). The information on sex can be found in Supplementary Table 1. Sex has also been considered in our manuscript. We did the sex-based analysis in our work regarding that females had higher incidence of the nCRT induced diarrhea than males.
Population characteristics	All 165 enrolled patients have provided written informed consent, and received the complete treatment course of nCRT (50Gy/25 fractions; concurrent capecitabine + irinotecan chemotherapy) and 1 cycle of interval chemotherapy (CAPIRI, capecitabine+ irinotecan). The dosage of irinotecan was determined by UGT1A1 genotype. The detailed demographic information can be found in Supplementary Table 1.
Recruitment	Our previous studies described the recruitment of this cohort in detail:<br>Zhu J, et al. Multicenter, Randomized, Phase III Trial of Neoadjuvant Chemoradiation With Capecitabine and Irinotecan Guided by UGT1A1 Status in Patients With Locally Advanced Rectal Cancer. Journal of clinical oncology 38, 4231-4239 (2020).<br><br>Eligible patients were aged 18-75 years old and diagnosed with clinical stage T3-4 and/or N+ rectal adenocarcinoma. The inclusion criteria included a Karnofsky performance status score ≥ 70, a UGT1A1 genotype of *1*1 or *1*28, adequate bone marrow function (a hemoglobin level ≥ 9 g/dL, neutrophil count ≥ 1,500/mL, and platelet count ≥ 100,000/mL), liver function (total bilirubin level < 1.5 times the upper limit of normal; albumin level > 30 g/L; and aspartate aminotransferase, alanine aminotransferase, and alkaline phosphatase levels < 2.5 times the upper limit of normal), and normal kidney function (creatinine concentration below the upper limit of normal)<br><br>Eligible patients were randomly allocated and received radiotherapy with concurrent capecitabine and irinotecan. The study protocol was approved by the central ethics committee of Fudan University Shanghai Cancer Center (Shanghai, China). All enrolled patients have provided informed consent and got free therapies as compensation.
Ethics oversight	The study protocol was approved by the central ethics committee of Fudan University Shanghai Cancer Center (Shanghai, China).

Note that full information on the approval of the study protocol must also be provided in the manuscript.

Field-specific reporting

Please select the one below that is the best fit for your research. If you are not sure, read the appropriate sections before making your selection.

[x] Life sciences   [ ] Behavioural & social sciences   [ ] Ecological, evolutionary & environmental sciences

For a reference copy of the document with all sections, see nature.com/documents/nr-reporting-summary-flat.pdf

Life sciences study design

All studies must disclose on these points even when the disclosure is negative.

Sample size	No statistical methods were used to calculate the sample size. Sample size was determined based on the number of serum sample collected from LARC patients in Fudan University Shanghai Cancer Center between July 2014 and January 2018. Totally, serum samples (n=743) of 165 LARC patients were collected.
Data exclusions	No data exclusion in this study.
Replication	Based on the stability and strict quality control (See methods and supplementary figure 11), the mass spectrometric measurement was carried out once for each patient's serum sample in this study. The following statistical experiments in Figure 1-5 were based on the metabolomics data from serum samples (n=743) of 165 LARC patients and performed independently.

Randomization	We would like to note that the participants was from a randomized Phase III trial and the experimental groups were randomly allocated.<br><br>In this study, the groups were divided by outcomes recorded in the trial, including therapeutic toxicities and treatment responses of nCRT. Those outcomes were blind before treatment. Serum samples for metabolomics study were randomly collected from this trial without any special design.<br><br>For all datasets, samples were assigned randomly to acquire metabolomics data using LC-MS.
Blinding	The investigators were blinded to group information during data collection. At the time of sample acquisition and processing, scientists were completely unaware of the sample group.

Reporting for specific materials, systems and methods

We require information from authors about some types of materials, experimental systems and methods used in many studies. Here, indicate whether each material, system or method listed is relevant to your study. If you are not sure if a list item applies to your research, read the appropriate section before selecting a response.

Materials & experimental systems

n/a	Involved in the study	
☒	☐	Antibodies
☒	☐	Eukaryotic cell lines
☒	☐	Palaeontology and archaeology
☒	☐	Animals and other organisms
☐	☒	Clinical data
☒	☐	Dual use research of concern

Methods

n/a	Involved in the study	
☒	☐	ChIP-seq
☒	☐	Flow cytometry
☒	☐	MRI-based neuroimaging

Clinical data

Policy information about clinical studies

All manuscripts should comply with the ICMJEguidelines for publication of clinical research and a completedCONSORT checklist must be included with all submissions.

Clinical trial registration	ClinicalTrials.gov identifier: NCT02605265
Study protocol	The protocol of this trial was provided in our previous work (https://ascopubs.org/doi/suppl/10.1200/JCO.20.01932).
Data collection	The LARC patients were collected from Fudan University Shanghai Cancer Center between July 2014 and January 2018.
Outcomes	Primary Outcome Measures :<br>Pathologic Complete Response (pCR) [Time Frame: Surgery scheduled 6-8 weeks after the end of chemoradiation]: pCR was defined as pathological T0N0M0 and all pCR statuses were evaluated by 2 independent pathologists. If their conclusions were inconsistent, it was evaluated again by a third pathologist.<br><br>Secondary Outcome Measures :<br>Overall Survival [Time Frame: From date of randomization until the date of death from any cause, assessed up to 10 years]<br>Disease-free Survival [Time Frame: From date of randomization until the date of first documented progression or date of death from any cause, whichever came first, assessed up to 10 years]<br>Local Control rate [Time Frame: From date of randomization until the date of first documented pelvic failure, assessed up to 10 years]<br>Number of participants with pelvic failure after surgery, evaluated using Kaplan-Meier Curve<br>Number of participants with chemoradiation-related adverse events as assessed by CTCAE v4.0 [Time Frame: Up to 2 years]<br>Surgical complications [Time Frame: Surgery scheduled 6-8 weeks after the end of chemotherapy]
